# Gut Microbiota-Mediated SN-38G Metabolism In Vitro: Effects of *Murraya koenigii* and *Ruta graveolens*

**DOI:** 10.3390/ijms27219582

**Published:** 2026-10-27

**Authors:** Torsten P. M. Scheithauer, Emanuele Olgiati, Elwin Verheij, Wieke van Butselaar, Michel Ossendrijver, Michelle van der Wurff, Frank H. J. Schuren

**Affiliations:** 1 Microbiology and Systems Biology, TNO The Netherlands Organization for Applied Scientific Research, 2333 BE Leiden, The Netherlands; torsten.scheithauer@tno.nl (T.P.M.S.); olgiatiemanuele98@gmail.com (E.O.); elwinverheij@kpnmail.nl (E.V.); wieke.vanbutselaar@outlook.com (W.v.B.); michel.ossendrijver@tno.nl (M.O.);; 2 Independent Researcher; 3 AVS Bio Netherlands B.V., Uppsalalaan 17, 3584 CT Utrecht, The Netherlands

**Keywords:** gut microbiota, in vitro models, drug metabolism, cancer therapy, GI complaints

## Abstract

The influence of the human microbiome on health and disease has been extensively studied over the past two decades. More recently, the microbiota has also been implicated in modulating the efficacy and toxicity of various drugs, including cancer therapies. This study focuses on irinotecan, a chemotherapeutic prodrug used in colorectal cancer treatment. Irinotecan is converted to the active metabolite SN-38, which is then glucuronidated in the liver to form the inactive metabolite SN-38G. Following biliary secretion into the intestine, microbial enzymes can cleave the glucuronide group and release SN-38, a process that may contribute to gastrointestinal toxicity. Human fecal microbiota converted SN-38G to SN-38 in vitro. Antibiotic treatment markedly reduced conversion, indicating that microbial activity accounted for most of the conversion under these conditions. In an exploratory screen, extracts from *Murraya koenigii* and *Ruta graveolens* reduced SN-38G conversion. Differences in community composition and beta-glucuronidase gene abundance were observed after extract exposure. These results show microbiota-dependent SN-38G conversion in vitro and identify compounds and plant extracts that delay or inhibit this conversion. Whether this effect is caused by direct inhibition of microbial beta-glucuronidase or by more generic microbiota effects is open for future research.

## 1. Introduction

The role of the human microbiome in health and disease has been intensively studied over the past 20 years, highlighting the significant importance of the gut microbiome in nearly all aspects of human health [1]. Driven by novel sequencing technologies, numerous studies have analyzed microbiome composition [2]. Although this approach has generated novel insights, revealing differences between the microbiomes of healthy individuals and those with various diseases, translating these data into a better understanding of the specific role of the microbiome in human health has proved challenging [3]. One striking observation is the enormous individual variation in microbiome composition, which complicates the correlation between microbiome composition and health outcomes [4]. Given the substantial functional overlap between microbial species, attention has shifted towards a more functional approach, focusing on the metabolites produced, active pathways, and detectable enzyme activities [5]. This shift has led to novel insights into the role of the microbiome in relation to the human host, largely independent of the specific microbiome composition.

More recently, the microbiota has been implicated in modulating the efficacy and toxicity of drugs, such as cancer therapies [6]. Here, we will focus on the drug irinotecan. This is one of the few cancer drugs for which the influence of the gut microbiome is somewhat understood. Irinotecan is a chemotherapeutic prodrug used for colorectal cancer. It is metabolically activated to 7-ethyl-10-hydroxycamptothecin (SN-38) by carboxylesterases in the liver or blood [7]. Only 2–5% of irinotecan is converted to SN-38 [8]. SN-38, in its active form, is inactivated by hepatic glucuronidation and excreted into the gut as SN-38 glucuronide (SN-38G) through biliary secretion [9]. Microbial beta-glucuronidases can hydrolyze the glucuronide group, releasing the active form SN-38 into the intestinal environment. This may lead to side effects of the drug [10].

Treatment with irinotecan can cause life-threatening diarrhea, which occurs sporadically even in patients in relatively good condition. However, not all patients suffer from this toxicity. According to several studies, the incidence of this side effect ranges from 11 to 36% [11]. Studies have shown a correlation between the degree of glucuronidation and the presence of severe diarrhea. The microbial beta-glucuronidases in the gut can reactivate SN-38G into SN-38. Previously, it has been shown that individual stool samples have variable capacities to hydrolyze SN-38G [12]. In the past, antibiotics were used to reduce irinotecan-induced toxicity. However, with this antibiotic treatment, gut microbes were depleted, and the protective functions of the microbiome could be lost [13].

Different mechanisms are known by which metabolites from irinotecan, produced by the gut microbiota, can damage human cells. For example, during DNA replication, DNA topoisomerase binds to the DNA and cuts the phosphate backbone to release some strain of the coiled DNA. The metabolite SN-38 can inhibit this replication process by stabilizing the DNA-enzyme intermediate. As a result, the DNA cannot ligate and continue with replication [14]. The stabilized DNA-enzyme intermediate collides with the replication fork, leading to irreversible double-strand DNA breakage and eventually apoptotic cell death [11]. Preventive strategies may reduce the toxic effects of irinotecan. One approach to prevent irinotecan toxicity would be to inhibit beta-glucuronidase activity during treatment.

Guthrie et al. (2019) described a bioinformatic approach (MicrobeFDT) to identify candidate compounds based on predicted relationships between microbial enzymes and 10,000 food and drug compounds [15]. We used MicrobeFDT to select compounds with a high probability of interfering with SN-38G metabolism and tested these compounds in our i-screen platform, which mimics the human gut microbiota in vitro. *Ruta graveolens* and *Murraya koenigii* were selected for extract testing because several compounds linked to these plants were present among the database candidates (Tables S3 and S4). Here, we present the results of this exploratory screening and identify candidates for further validation.

## 2. Results

In this study, we aimed to identify compounds that could reduce microbiota-mediated conversion of SN-38G to SN-38. We identified candidate compounds through computational analysis and used MicrobeFDT to select compounds with a high probability of interfering with SN-38G metabolism. These candidates were tested on our i-screen platform, which mimics the human gut microbiota in vitro. The screen was performed with one sample per condition and did not measure beta-glucuronidase activity directly. The findings are therefore interpreted as exploratory and require independent validation.

### 2.1. Platform Development

Studying microbiome-driven drug metabolism in humans is very challenging; therefore, we developed an in vitro platform to mimic the processes occurring in the human colon, simplifying such studies. We demonstrated that on this platform colon microbiota metabolizes compounds such as sulfasalazine, sulfinpyrazone, sulindac, nizatidine, and risperidone, all known to be metabolized in vivo as well [16]. We further developed this platform to study the metabolism of SN-38G, approaching this metabolism not only from a qualitative perspective, as in our earlier work, but also focusing on quantitative analysis.

Initially, we did not know how quickly conversion might occur, so in our first experiment, we collected samples at 19 min, 25 min, 1 h, and 6 h. Results showed little change compared with baseline (*t* = 0 h) during the first hour. However, after 6 h, almost all SN-38G had been converted. Consequently, we conducted a new experiment with samples taken every hour. Results from this analysis are shown in Figure 1. The conversion of SN-38G into SN-38 began slowly after 2 to 3 h under the selected experimental conditions, accelerated between 4 and 5 h, and after 6 h no SN-38G was detectable (Figure 1A). In the triplicate assay wells, both SN-38G and SN-38 differed from baseline from 4 h onward. These results show that the pooled fecal microbiota converted SN-38G under the conditions used here.

Adding clindamycin markedly reduced SN-38G conversion (Figure 1B). SN-38 differed from baseline at 2, 5, 6, 7, and 8 h, with a minor increase in abundance. Even after 8 h, SN-38G was far more abundant than SN-38, indicating that microbial activity contributed substantially to the conversion of SN-38G to SN-38. Clindamycin is a lincosamide antibiotic that primarily targets Gram-positive bacteria [17]. Incubating SN-38G under the same conditions but without microbiota resulted in a slight decrease to approximately 90% of the starting amount, indicating that some nonmicrobial conversion also occurred. These results suggest that the gut microbiota was responsible for most, but not all, SN-38G conversion under these conditions.

A 100× diluted pre-culture of the pooled fecal inoculum was incubated with 50 µM SN-38G. The percentages of SN-38G and SN-38 were calculated from their combined concentration at each time point. (A) SN-38G was no longer detectable after 6 h. (B) Clindamycin markedly delayed SN-38G conversion. The experiment was performed in triplicate assay wells using the same pooled inoculum. A two-way ANOVA was used in the original analysis, with each time point compared with the baseline. Letters indicate *p* < 0.05 for SN-38G (a) or SN-38 (b) relative to the baseline. Abbreviations: 7-ethyl-10-hydroxycamptothecin (SN-38), SN-38 glucuronide (SN-38G).

### 2.2. Selection of Candidate Compounds

The MicrobeFDT database was set up locally according to the manual published on GitHub [15]. Using the Cypher query language, we searched for compounds with structural similarity to SN-38G. Compounds with a relationship score of at least 0.57 were selected, resulting in 501 compounds (Table S2). A second search identified compounds known to be metabolized by β-glucuronidase, yielding 58 compounds, 15 of which also had a substructure similarity with a relationship score of 0.57 or higher (Table S3). Of the total 544 compounds, 13 were selected based on their availability (Table 1). This selection included a variety of drugs and non-drugs, compounds known and unknown to be metabolized by β-glucuronidase, and varying relationship scores and origins.

Additionally, the source/origin of all 544 compounds was determined. The ornamental plant *Ruta graveolens* and the curry leaf tree *Murraya koenigii* were the origins of 22 and 37 compounds, respectively, within the 501-compound selection, with a relationship score above 0.57 (Tables S4 and S5). In total, 18 compounds and extracts were tested for their effects on SN-38G conversion (Table 1).

### 2.3. Screening of Compounds Affecting SN-38G Conversion

Selected compounds and extracts were incubated on the i-screen platform, with medium samples taken at the 6 h mark. To explore concentration effects, two concentrations were tested (Figure 2). During the screening, 11 of the 18 test compounds and extracts did not reduce conversion below 50%, resulting in more than 50% of SN-38G being converted to SN-38 (Figure 2A). In contrast, the antibiotics strongly reduced conversion, with a maximum of 38% of SN-38G converted to SN-38 in the presence of the broad-spectrum antibiotic ampicillin (Figure 2B) [18]. The fraction of SN-38G converted to SN-38 under anaerobic conditions may be due to spontaneous conversion or the activity of enzymes remaining after bacterial cell inactivation. Because the screening was performed with one sample per condition, no statistical testing was performed, and these results are exploratory.

In total, 7 of the 18 tested compounds and extracts reduced SN-38G conversion in the screening experiment (Figure 2C). Different conversion values were observed at the two tested concentrations for several candidates. For example, berberine reduced the amount of SN-38 in the untreated control to 49% at 0.05 mM and 32% at 0.15 mM. Berberine, a compound used in traditional medicine, has antibacterial properties and may reduce bacterial activity [19]; direct inhibition of beta-glucuronidase was not tested. The strongest effect in this screen was observed with Ruta graveolens ethanol extract (RGE). At the two tested concentrations, RGE reduced conversion of SN-38G to SN-38 to 19% and 13%, respectively. These observations require independent validation and do not distinguish selective enzyme inhibition from broader effects on microbial activity.

### 2.4. Individual Variation

A first analysis targeted individual variation in SN-38G metabolism by using 10 individual microbiota donors, all of which were exposed to clindamycin and *Murraya koenigii* ethanol extract (one of the most active lead extracts from the first screen) (Figure 3). Clear differences were observed, especially in the levels of SN-38G metabolism after 3 h, the first point measured in this experiment, with SN-38G levels varying between 25% and 70%, as well as in the rate of metabolism in the following hours (Figure 3A). All individuals converted SN-38G to SN-38 completely within 24 h of incubation. In the analysis shown in Figure 3, both time and individual donor were associated with the observed variation (*p* < 0.0001). When SN-38G was incubated under the same conditions but without microbiota, only 12% was converted to SN-38 within 24 h. Differences in microbiota composition may contribute to the observed variation in conversion, but if and how this affects toxicity was not assessed in this study.

Incubations with clindamycin and *Murraya koenigii* ethanol extract were performed for all individuals (Figure 3B and Figure 3C, respectively). Both the levels of SN-38G after 24 h and the effects of clindamycin and *Murraya koenigii* extract differed among individuals. Conversion of SN-38G continued up to 24 h despite antibiotic and extract treatment, possibly because of residual enzymatic activity from inactivated bacteria or other mechanisms. Both treatments delayed conversion, leaving up to 39% of SN-38G unprocessed. After calculating the area under the curve (AUC) for each individual and comparing the treatments, both clindamycin and the *Murraya koenigii* extract increased the AUC (Figure 3D). These results show that the extract delayed SN-38G conversion across the donor samples tested, but further replicated experiments are needed before its effect can be generalized.

### 2.5. Gut Microbiota Composition

To gain further insight into changes in the gut microbiota, we analyzed the samples using metagenomic sequencing (Figure 4 and Figure S1). Both antibiotics, amoxicillin and clindamycin, markedly reduced alpha diversity, leaving only a few bacterial species, primarily *Escherichia coli*, in the i-screen samples and separating the antibiotic-driven results from the other controls, compounds, and extracts in the PCA plot (Figure S1). These observations are consistent with broad antimicrobial effects on the microbial community and may help explain the reduced SN-38G conversion observed with antibiotics.

Next, we investigated the effects of SN-38G on the gut microbiota, focusing on the extracts and the two controls, SN-38G and the DMSO vehicle control. This focus was based on the effects of the extracts on SN-38G conversion and microbial composition compared with the other compounds. Alpha diversity in the SN-38G condition was similar to that observed with the DMSO vehicle control (Figure 4B). No obvious shifts in composition were observed after 24 h of incubation with SN-38G compared with the DMSO control, and the samples clustered close to each other in the PCA plot (Figure 4A). Given the limited metagenomic replication, these observations are descriptive. Within this dataset, there was no clear evidence that SN-38G alone caused a large shift in community composition over 24 h.

Several compounds reduced the rate of SN-38G conversion, with the largest effects in the screening experiment observed for the herbal extracts. Most conditions showed alpha diversity values similar to those of the DMSO control. The herbal extract conditions, however, showed lower diversity at the higher tested concentrations (Figure 4A,B). In the PCA analysis, extract-treated samples were separated from the controls, particularly at the highest concentration tested (Figure 4A). Species abundance also differed in the extract-treated samples (Figure 4C,D), including a higher relative abundance of *Lachnospira eligens* in this dataset. However, each extract concentration was represented by only one sample, so these observations cannot establish a reproducible dose response. Replicated experiments are needed to determine whether these community changes are reproducible and which components of the extracts are responsible.

Lastly, we investigated the beta-glucuronidase gene abundance after 24 h of incubation using metagenomic sequencing data (Figure 4E). Compared with the DMSO control, all SN-38G-treated samples showed a higher abundance of this gene. However, extract-treated samples did show lower abundance of this gene than the SN-38G control sample. Due to the low number of replicates, these results should be considered as exploratory. Metagenomic gene abundance also does not demonstrate gene expression or enzyme activity, and beta-glucuronidase activity was not measured directly.

## 3. Discussion

The gut microbiota is involved in metabolizing various orally administered drugs, as well as some drugs administered systemically and excreted into the intestine [15]. Irinotecan falls into this latter category [7]. It is converted to the active metabolite SN-38, which is subsequently glucuronidated to SN-38G and excreted into the gut. Microbial beta-glucuronidases can convert SN-38G back to SN-38, a pathway linked to gastrointestinal toxicity. In this study, we first tested whether the gut microbiota could convert SN-38G to SN-38. Next, we explored whether selected compounds and plant extracts could reduce this conversion. The assay quantified SN-38G and SN-38 but did not directly measure beta-glucuronidase activity.

To investigate whether selected compounds can influence the conversion of SN-38G to SN-38, we conducted an i-screen assay. In this assay, selected compounds were added to a microbiota culture containing SN-38G, and the conversion process was analyzed. TNO’s i-screen is a high-throughput fermentation model that utilizes human fecal microbiota to assess the effects of gut microbiota on orally administered or systemically excreted compounds [16]. Using the MicrobeFDT database, over 500 compounds were identified as candidates, from which 13 single compounds and five plant extracts were selected for experimental testing together with seven antibiotics. SN-38G and SN-38 levels were analyzed using LC-MS. Eleven of the 18 non-antibiotic candidates did not reduce SN-38G conversion below 50% within 6 h of in vitro incubation. Because the screening was performed with one sample per condition, these results are exploratory.

At baseline, 7% of SN-38G was already detected as SN-38, which may partly reflect the time between compound addition and sampling. Overall, microbial conversion occurred rapidly. We also observed some conversion of SN-38G in the absence of microbiota, suggesting that the compound is not fully stable under the assay conditions. The contribution of anaerobic conditions, handling, or other nonmicrobial factors was not determined. This background conversion should therefore be considered when interpreting the effects of microbial or antimicrobial treatments.

SN-38G was rapidly converted by the microbiota, marking an important first step in this research. Here, we present the results for seven compounds and extracts that reduced conversion in the initial screen, providing preliminary information on how these candidates may alter SN-38G conversion in vitro. More controlled experiments are needed for firm conclusions. The fecal inocula used in this study were obtained from healthy adult donors. The microbiota of patients receiving irinotecan may differ because of the underlying disease, chemotherapy, concomitant medication, antibiotic exposure, diet, and other clinical factors. Conversion rates and responses to the tested compounds may therefore differ in patients. Previous work with the i-screen platform showed correspondence between in vitro and in vivo responses in a dietary-fiber setting [20,21], but this does not establish translational value for irinotecan metabolism. Studies using patient-derived microbiota are needed before the clinical relevance of the model can be assessed.

Longer incubation times might alter the observed effects, but SN-38G conversion occurred rapidly under the conditions used here. The present results are not sufficient to support clinical testing of the candidate compounds or extracts. The effects observed for the leading candidates should first be reproduced in independent experiments and examined with direct beta-glucuronidase activity assays and measurements of microbial growth or viability. For the plant extracts, chemical characterization, batch reproducibility, safety, potential drug interactions, and preclinical validation are also needed before clinical relevance can be assessed.

All seven antibiotics reduced SN-38G conversion, but 23% to 38% of SN-38G was still converted after 6 h. This residual conversion might be due to spontaneous conversion of SN-38G, bacteria that are resistant to the tested antibiotics, or enzymes still present despite bacterial inactivation. Antibiotic treatment also caused broad changes in the microbiota composition. These results show that reduced SN-38G conversion can arise from general suppression of microbial activity. Because microbial growth or viability was not measured alongside the candidate compounds and extracts, a similar nonspecific mechanism cannot be excluded for those treatments.

The single compound berberine, obtained from *Berberis vulgaris* [22], showed a reduction in SN-38G conversion similar to that observed with the *Murraya koenigii* extract in the screening experiment. The lowest conversion values were observed with the *Ruta graveolens* ethanol extract. At the highest tested concentration, only 13% of SN-38 was measured, comparable to the baseline sample. *Ruta graveolens* has reported antibacterial activity [23], which could contribute to the observed effect. However, the crude extracts were not chemically characterized in this study, and the concentrations used in the screen were relatively high. The active constituents, mechanism, exposure relevance, safety, and potential interactions with irinotecan remain unknown. *Ruta graveolens* should therefore be considered an exploratory lead for further analytical and preclinical work rather than a candidate for clinical use, on the basis of the present data.

We further examined the composition of the fecal microbiota after incubation with the test compounds. As expected, antibiotics caused large changes in bacterial composition and diversity, which has been shown in many studies and is related to host health [24,25]. These changes were more pronounced than those observed with SN-38G itself, which remained close to vehicle control in the available metagenomic data. The plant extracts were also accompanied by changes in community composition. This is relevant to the conversion results because reduced SN-38G conversion could result from broader changes in microbial abundance or activity rather than selective beta-glucuronidase inhibition. Several taxonomic differences were observed after extract exposure, including changes in *Lachnospira*-related taxa [26]. *Lachnospira* is a genus known for its pectin degrading activity, which suggests that pectin in plant extracts may play a role in the observed results [27]. Pectins are naturally occurring fibers in plant cell walls [28] and have been shown to support gut microbiome function as well as gut health [27,29], but they have also been shown to support irinotecan therapy in patients [30]. Since the extract conditions had limited replication, these observations require confirmation in independently replicated experiments.

The experiments show that human fecal microbiota can convert SN-38G to SN-38 in vitro and that antibiotics, berberine, and extracts from *Murraya koenigii* and *Ruta graveolens* reduced this conversion under the conditions tested. The candidate screen and metagenomic analyses were exploratory, and direct inhibition of microbial beta-glucuronidase was not demonstrated. Independent replication, measurements of microbial growth and enzyme activity, chemical characterization of plant extracts, and testing in patient-derived or preclinical models are needed before clinical relevance can be assessed.

The MicrobeFDT database can be combined with wet-lab experiments using the i-screen technique to prioritize compounds for further testing. Here, we used the database to identify compounds with structural relationships to SN-38G before testing them in the i-screen model. There are other cases in which a similar approach could be useful. For example, the primary medication for treating Parkinson’s disease is levodopa (L-dopa) [31]. The gut microbiota can contribute to L-dopa decarboxylation and dopamine formation [32], and differences in microbial activity may contribute to variability in treatment response [33]. In cases where microbial metabolism influences drug response, computationally selected candidates can be tested experimentally, but replicated validation is still required.

## 4. Materials and Methods

### 4.1. Materials

SN-38 Glucuronide (trifluoroacetate salt, SN-38G) was obtained from Cayman Chemical, Ann Arbor, MI, USA. SN-38 (NK012) was obtained from MedChemExpress, Monmouth Junction, NJ, USA. The effects of the test compounds on SN-38G conversion were tested using several compounds (Table 1). Most of the compounds were obtained from Sigma Aldrich (Saint Louis, MO, USA), unless otherwise specified.

### 4.2. In Vitro Fermentation

The fermentation experiments followed previously published i-screen procedures, with minor modifications [16,34]. Fecal samples were collected in anaerobic jars and handled within 24 h. Fecal material was homogenized in an anaerobic chamber in SIEM (Table 2). After, homogenization glycerol was added (to reach a 12% final concentration), and the sample was homogenized once more. The resulting slurry was distributed over multiple tubes and frozen at −80 °C in 12% glycerol. Before use, the frozen human colon microbiota inoculum was precultured overnight in SIEM at 37 °C and 300 rpm under anaerobic conditions [35]. The SIEM and vitamin compositions are provided in Table 2 and Table 3. Components were obtained from Tritium Microbiology (Eindhoven, The Netherlands), and prepared SIEM was kept at 4 °C in an anaerobic jar for no longer than 48 h. The screening experiments used pooled microbiota preparation containing fecal material from 6 healthy adult donors. The individual-donor experiment was performed with 10 individual samples from healthy adults next to the pooled sample used in the screening experiment. There was no overlap between the 6 pooled samples and the 10 individual samples.

Stock concentrations of test compounds (dissolved in DMSO) were further diluted in modified SIEM to final concentrations of 50, 150 and 500 µM in the screening platform (96 deep-well plates with 2 mL volume per well). The final DMSO concentration was 1% in all incubations. The precultured pooled human colon microbiota (0.1%, *v*/*v*) were incubated for 24 h (pH 5.8; 300 rpm) in fully anaerobic chambers (A45 Scientific Anaerobic Workstation; Don Whitley, Bingley, UK) at 37 °C under 80% nitrogen, 10% carbon dioxide and 10% hydrogen. The samples were split through centrifugation (see below) into a supernatant-containing medium used for subsequent LC-MS analysis and the pellet with microbes used for sequencing analysis. Incubations with only microbiota in SIEM and incubations with only SIEM served as negative background controls for the LC-MS analyses. Incubations with test compounds in SIEM (without microbiota) served as a negative control for microbiota-based metabolism to determine compound stability or nonspecific degradation of the test compounds due to culture conditions during the assay. A total of 50 µL of each sample was transferred to a new plate kept on ice. Subsequently, 200 µL of acetonitrile was added to each well in the flow cabinet. The plate was then centrifuged at 3000 rpm for 10 min. The supernatants were transferred to a new plate and centrifuged again at 3000 rpm for 10 min. Finally, the resulting supernatants were transferred to another new plate.

### 4.3. Selection of Potential Inhibitors with the MicrobeFDT Database

The MicrobeFDT database was set up locally according to the manual published on GitHub [15]. Using the Cypher query language, compounds with structural similarity to SN-38G were searched. Compounds with a relationship score of at least 0.57 were selected, resulting in 501 compounds (Table S2). A second search identified 58 compounds known to be metabolized by beta-glucuronidase (Table S3), 15 of which also had a substructure-similarity relationship score of at least 0.57. Accounting for this overlap yielded 544 unique compounds. Thirteen single compounds were selected based on availability. Source information was then examined for the 501 structurally related compounds. *Ruta graveolens* and *Murraya koenigii* were associated with 22 and 37 of these compounds, respectively (Tables S4 and S5), which provided the rationale for testing extracts from both plants. In total, 18 single compounds and plant extracts were included in the candidate screen (Table 1).

### 4.4. Screening Selected Compounds

The selected single compounds (Table 1) were dissolved in DMSO and diluted in the 2 mL i-screen wells to final concentrations of 150 or 500 µM. Water and ethanol extracts were prepared from dried *Murraya koenigii* (Vitabron) and *Ruta graveolens* (Jacob Hooy). Plant materials were obtained from a drugstore selling these materials. Extracts were made from these materials as herein described. For the water extracts, 250 mg dried plant material was added to 5 mL Milli-Q water and incubated for 30 min at 90 °C. For the ethanol extracts, the dried material was ground with a mortar and pestle, after which 250 mg was added to 4 mL ethanol and heated until the volume was reduced by half. The extract was then diluted to a final ethanol concentration of 30%. A commercial *Ruta graveolens* extract (Natura Sanat B.V.) was also tested. This extract is a 30% ethanol in water extract containing 100 mg of plant material per ml. Extracts were added to the i-screen wells to final concentrations of 3% and 10%. The antibiotics listed in Table 1 were added at a final concentration of 10 µg/mL. The prepared and commercial plant extracts were used as crude extracts and were not chemically characterized in this study.

For the screening experiments, the i-screen was inoculated with a standardized adult fecal microbiota preparation. Fecal samples were collected from six healthy adult volunteers. Donors were described as Caucasian, followed a European lifestyle and diet, and had not used antibiotics during the preceding three months. Collection was approved by TNO’s internal ethical review board. The inoculum was diluted either 100-fold in SIEM or 50-fold in double-strength SIEM. Wells containing single compounds, antibiotics or controls received 1455 µL of the 100-fold dilution, whereas extract wells received 750 µL of the 50-fold dilution. Incubations were started by adding 15 µL SN-38G (5 mM in 50% DMSO). Samples of 50 µL were collected at 0, 19, 25 and 45 min and at 1, 2, 3, 4 and 6 h. Each sample was transferred to a 1 mL plate and kept on ice before 200 µL acetonitrile was added in a flow cabinet. Samples were centrifuged at 3000 rpm for 10 min, the supernatant was transferred to a new plate, and this centrifugation step was repeated. Supernatants were stored at −80 °C until LC-MS analysis.

### 4.5. LC-MS Analysis

Samples from the i-screen model were extracted with 4 volumes of acetonitrile. After centrifugation, the supernatants were transferred to autosampler vials. LC-MS analysis of SN-38 and SN-38G was done using an Acquity H-Class UPLC system (Waters, Milford, MA, USA) and a high-resolution mass spectrometer (Q-Exactive, Thermo, Waltham, MA, USA).

The column used was an HSS-T3 (Waters) at 40 °C, and mobile phases A and B consisted of 0.1% formic acid in water and acetonitrile respectively. The flow rate was 0.5 mL/min, and the gradient used was 0% B at 0 to 1 min, 100% B at 5 to 5.9 min, followed by 0% B at 6 to 7 min.

The mass spectrometer was operated in positive ion mode at a resolution of 17.500. Data were acquired by scanning from *m*/*z* 100 to 1200. Quantification of SN-38 and SN-38G was done by integrating the M + H+ ions with a 5 mmu window.

### 4.6. Sequencing of Microbiota

Genomic DNA isolation was performed as earlier described [36]. Shotgun metagenomic libraries were prepared with the Illumina DNA Prep protocol (Illumina DNA Prep Reference Guide, 1000000025416v10, San Diego, CA, USA). DNA concentrations were normalized across samples before tagmentation and cleanup.

Indexed i5 and i7 adapters were added during PCR amplification. The amplified libraries were then cleaned and pooled.

Sequencing was performed on an Illumina NextSeq instrument using NextSeq V3 chemistry.

### 4.7. Sequence Analysis

Raw sequence data and metadata are available through the ENA database under accession PRJEB113194. Sequence analysis, including host filtering, quality control and taxonomic profiling, was performed with the bioBakery 3 workflow [37]. KneadData version 0.10.0 was used for preprocessing, and MetaPhlAn 3.0 with the ChocoPhlAn database version mpa_v31_CHOCOPhlAn_2010901 was used for species-level taxonomic profiling. Taxonomic tables were imported into phyloseq [38], and taxa were filtered by prevalence and relative abundance as described below [36].

Taxa were filtered using a prevalence threshold of 0.1 and a relative abundance threshold of 0.001 to remove low-abundance features [39,40]. This reduced the number of taxa from 124 to 49. PCA plots before and after filtering were compared to check whether filtering substantially changed the overall sample distribution. A centered log-ratio (CLR) transformation was then applied before ordination to account for the compositional nature of the metagenomic data [41]. Sequencing data are available in ENA under accession PRJEB113194.

Figures were prepared with ggplot2 version 3.3 [42]. PCA and redundancy analysis (RDA) were performed with vegan version 2.5-7 [43]. Shannon alpha diversity was also calculated with vegan. Relative abundance heatmaps with hierarchical clustering were generated with pheatmap version 1.0.12.

PERMANOVA was performed with the adonis2 function in vegan [43] using 999 permutations and compound as the grouping factor. Euclidean distances were calculated from the transformed taxonomic data in phyloseq [38]. Because several treatment conditions were represented by a single sample, the PERMANOVA results were treated as exploratory and were not used as independent evidence of reproducible group differences.

### 4.8. Functional Analysis

Metagenomic functional profiles were generated with HUMAnN3 within the bioBakery 3 workflow [37]. HUMAnN3 maps reads to taxonomic references using MetaPhlAn3, aligns unmapped reads against the ChocoPhlAn pangenome and performs translated searches against UniRef90. Functional annotations were regrouped into enzyme commission categories and normalized for comparison across samples. Beta-glucuronidase gene abundance was visualized with ggplot2. Differential abundance of pathway and enzyme profiles was explored with limma [44], with visualization using EnhancedVolcano and pheatmap. Because the extract-treated conditions had limited replication, these comparisons were used only to explore possible differences and were not treated as evidence of a reproducible treatment effect.

### 4.9. Statistical Analysis

The percentage of SN-38G and SN-38 was calculated from their combined concentration at each time point. The time-course experiment shown in Figure 1 was performed in triplicate assay wells using the pooled inoculum; these wells are technical assay replicates and not independent donor-level biological replicates. Figure 1 was analyzed with a two-way ANOVA, with time points compared with the baseline. The candidate screen shown in Figure 2 contained one sample per condition, and no statistical testing was performed. For the individual-donor experiment in Figure 3, the donor was the biological experimental unit, and serial measurements were obtained within each donor over time. AUC values were calculated for each donor and treatment using GraphPad Prism version 9.1.2.

For the metagenomic extract conditions, each concentration was represented by a single sample. Samples from different concentrations were grouped for an exploratory DESeq2 analysis [45]. Concentrations are treatment levels rather than independent biological replicates, so this grouping does not provide biological replication. The resulting differential abundance comparisons were used only as exploratory analyses.

## Figures and Tables

**Figure 1:**
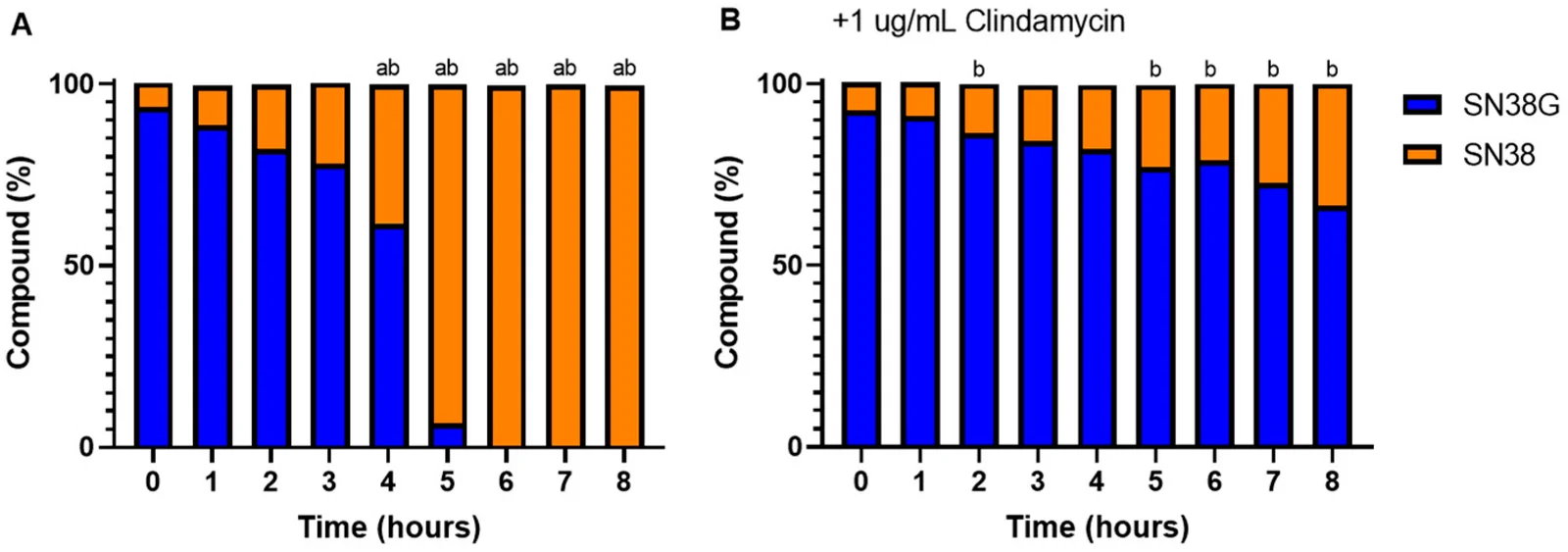
Time-dependent conversion of SN-38G by pooled fecal microbiota in vitro. (**A**) results without antibiotic, (**B**) results in the presence of Clindamycin.The raw data that form the basis for this figure were generated in triplicate and are available as Table S1.

**Figure 2:**
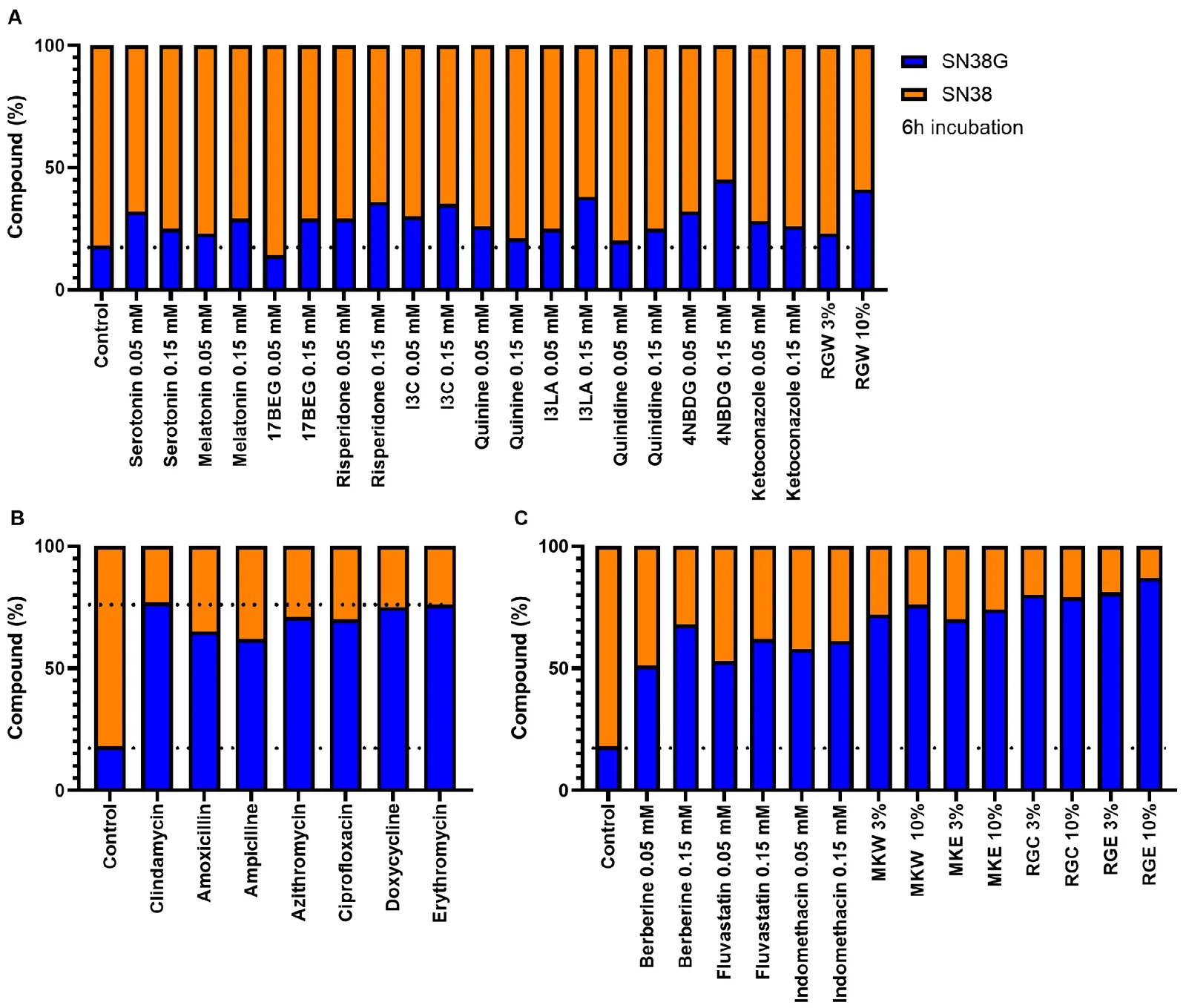
Metabolism of SN-38G by fecal microbiota in an in vitro setting. (**A**) Most test compounds did not reduce the conversion of SN-38G below 50%. Pooled fecal samples were used for the fermentation model. A 100x diluted pre-culture of fecal samples and 0.05 or 0.15 mM test compounds were combined with 50 μM SN-38G. The percentage of metabolized SN-38G was calculated from the total concentration of SN-38G and SN-38 at each time point. (**B**) Antibiotic treatment reduced SN-38G conversion. (**C**) Several compounds and extracts reduced SN-38G conversion. The screening was performed with a single replicate per condition, and no statistical testing was performed; the data are therefore descriptive and exploratory. Abbreviations: 7-ethyl-10-hydroxycamptothecin (SN-38), SN-38 glucuronide (SN-38G), 17β-Estradiol glucuronide (17BEG), Indole-3-carbinol (I3C), Indole-3-lactic acid (I3LA), 4-Nitrophenyl-β-D-glucuronide (4NBDG), *Ruta graveolens* water, ethanol, or commercial extract (RGW, RGE, or RGC), and *Murraya koenigii* water or ethanol extract (MKW or MKE). The raw data which form the basis for this figure are available as Table S6.

**Figure 3:**
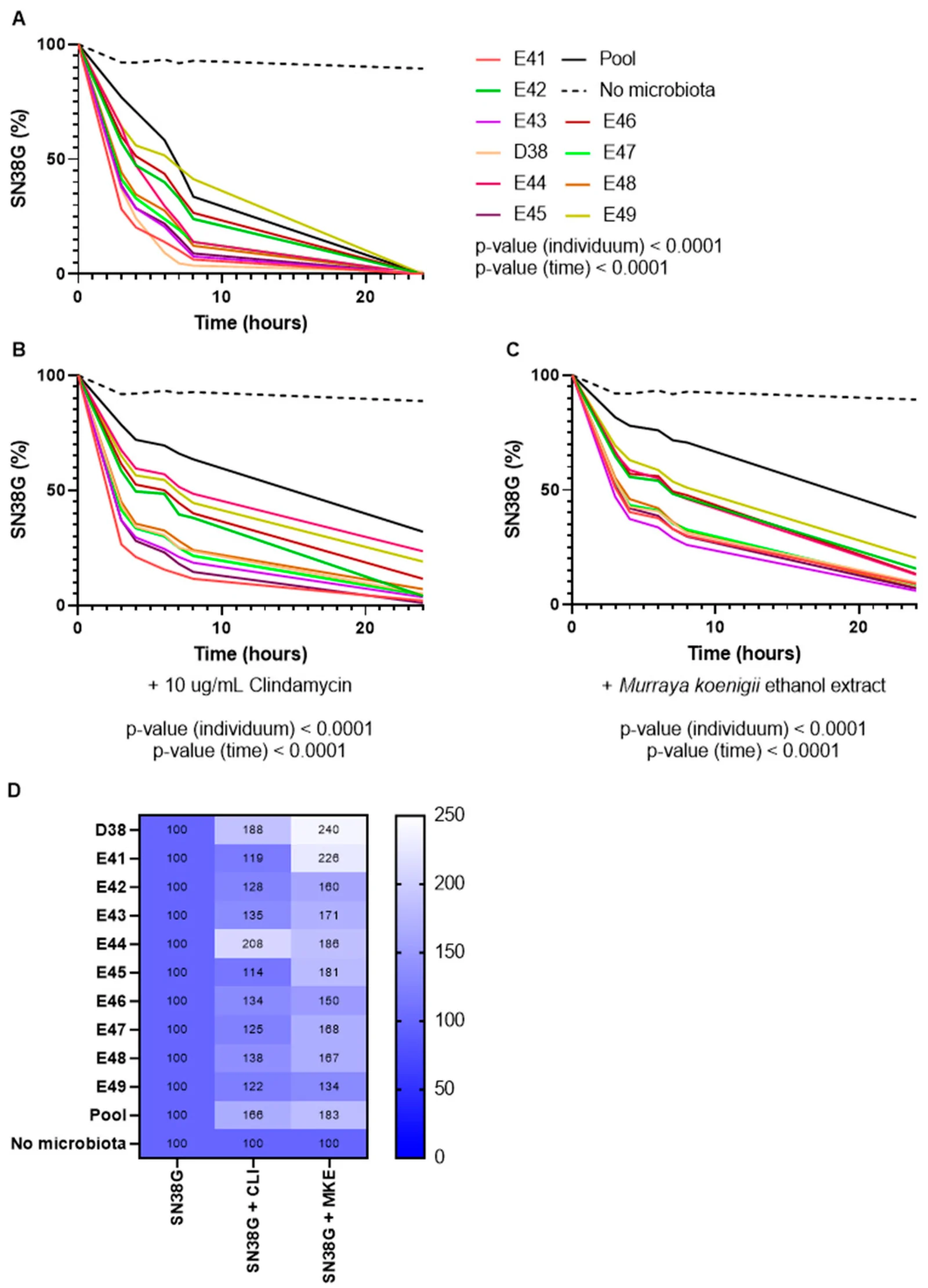
Inter-individual variation in SN-38G conversion. (**A**) SN-38G conversion was measured for 24 h in microbiota from 10 individual donors, the pooled fecal inoculum and a no-microbiota control. (**B**) Clindamycin delayed SN-38G conversion. (**C**) *Murraya koenigii* ethanol extract (MKE) also delayed conversion. (**D**) AUC values were calculated for each donor and treatment; higher AUC values indicate that more SN-38G remained during the incubation. The donor was the biological experimental unit. Abbreviations: 7-ethyl-10-hydroxycamptothecin (SN-38), SN-38 glucuronide (SN-38G). Statistical analyses were performed with 2-way repeated measures ANOVA, with time and individual donor as factors, applying the Geisser–Greenhouse correction. Raw data used for this figure are available in Supplementary Table S7.

**Figure 4:**
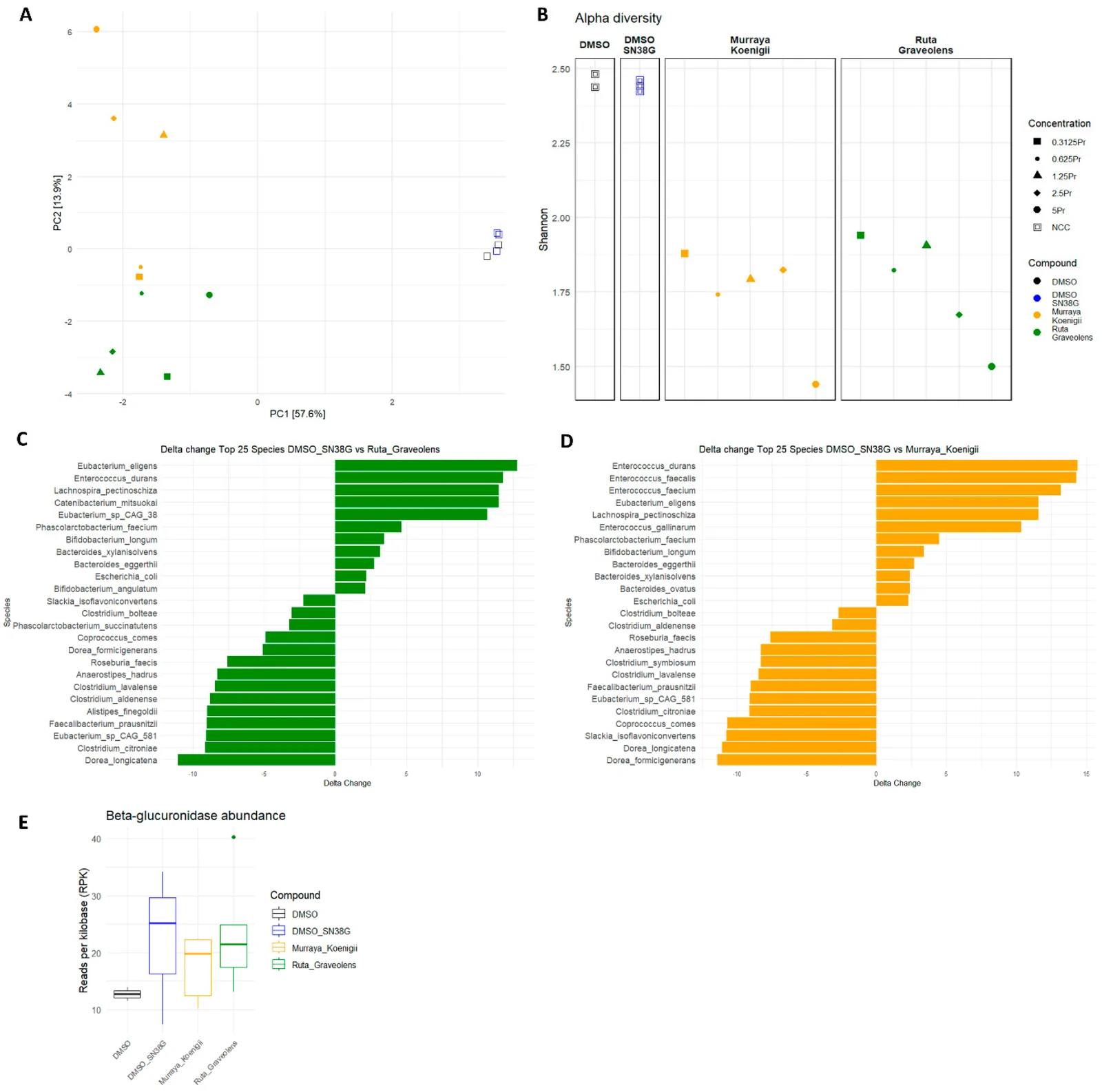
Effects of the tested extracts on fecal microbiota composition in vitro. Fecal microbiota was incubated for 24 h with the indicated conditions and analyzed by metagenomic sequencing. (**A**) Principal component analysis of community composition. (**B**) Shannon alpha diversity across control, SN-38G and extract-treated samples. (**C**) Changes in the relative abundance of the 25 displayed species for *Ruta graveolens*-treated samples compared with the DMSO/SN-38G control. (**D**) Corresponding species-level changes for *Murraya koenigii*-treated samples. (**E**) Metagenomic beta-glucuronidase gene abundance. Extract-treated conditions were represented by one sample per concentration; DMSO and SN-38G conditions contained n = 2 and n = 3 samples, respectively. These analyses are exploratory. Legend for (**A**,**B**) is shown on the right side of (**B**).

**Table 1:** Overview of tested compounds.

#	Others	Supplier
1	Quinine	Sigma-Aldrich, Zwijndrecht, The Netherlands
2	Quinidine	Sigma-Aldrich, Zwijndrecht, The Netherlands
3	Indomethacin	Sigma-Aldrich, Zwijndrecht, The Netherlands
4	Fluvastatin	Toronto Research Chemicals, Vaughn, Canada
5	Berberine	Sigma-Aldrich, Zwijndrecht, The Netherlands
6	Indole-3-lactic acid	Sigma-Aldrich, Zwijndrecht, The Netherlands
7	Indole-3-carbinol	Sigma-Aldrich, Zwijndrecht, The Netherlands
8	Ketoconazole (2R,4S)	Sigma-Aldrich, Zwijndrecht, The Netherlands
9	Risperidone	Sigma-Aldrich, Zwijndrecht, The Netherlands
10	4-Nitrophenyl-β-D-glucuronide	Sigma-Aldrich, Zwijndrecht, The Netherlands
11	17β-Estradiol glucuronide	Sigma-Aldrich, Zwijndrecht, The Netherlands
12	Serotonin	Sigma-Aldrich, Zwijndrecht, The Netherlands
13	Melatonin	VWR International, Boxmeer, The Netherlands
14	*Murraya* koenigii, also known as curry leaf, dried plant, water extraction (MKW)	Vitabron, Veenendaal, The Netherlands
15	*Murraya* koenigii, also known as curry leaf, dried plant, ethanol extraction (MKE)	Vitabron, Veenendaal, The Netherlands
16	*Ruta graveolens,* dried plant, water extraction (RGW)	Jacob Hooy, Limmen, The Netherlands
17	*Ruta graveolens,* dried plant, ethanol extraction (RGE)	Jacob Hooy, Limmen, The Netherlands
18	*Ruta graveolens,* commercial extract (RGC)	Natura Sanat B.V. Harderwijk, The Netherlands
**#**	**Antibiotics**	**Supplier**
19	Amoxicillin (AMX)	Sigma-Aldrich, Zwijndrecht, The Netherlands
20	Azithromycin (AZM)	Sigma-Aldrich, Zwijndrecht, The Netherlands
21	Ciprofloxacin (CIP)	Sigma-Aldrich, Zwijndrecht, The Netherlands
22	Clindamycin (CIJ)	Sigma-Aldrich, Zwijndrecht, The Netherlands
23	Doxycycline (DOX)	Sigma-Aldrich, Zwijndrecht, The Netherlands
24	Erythromycin (ERY)	Sigma-Aldrich, Zwijndrecht, The Netherlands
25	Ampicillin (AMP)	Sigma-Aldrich, Zwijndrecht, The Netherlands

**Table 2:** Media composition for anaerobic fermentation. All components were obtained from Tritium Microbiology (The Netherlands).

Compound	Concentration
sodium chloride (NaCl)	4.5 g/L
potassium hydrogen phosphate (K_2_HPO_4_)	2.5 g/L
calcium chloride dihydrate (CaCl_2_ · 2H_2_O)	0.45 g/L
magnesium sulfate heptahydrate (MgSO_4_ · 7H_2_O)	0.5 g/L
ferrous sulfate heptahydrate (FeSO_4_ · 7H_2_O)	0.005 g/L
Ox bile	0.05 g/L
hemin	0.01 g/L
cystein	0.4 g/L
pectin	0.6 g/L
xylan	0.6 g/L
arabinogalactan	0.6 g/L
amylopectin	0.6 g/L
starch	5 g/L
Tween 80	2 mL/L
bactopeptone	3 g/L
casein	3 g/L
vitamin mixture (see Table 3)	1 mL

**Table 3:** Vitamin mixture composition for anaerobic fermentation. All components were obtained from Tritium Microbiology (The Netherlands).

Compound	Concentration
menadione	1 mg/L
D-biotin	2 mg/L
vitamin B-12	0.5 mg/L
pantothenate	10 mg/L
nicotinamide	5 mg/L
para-aminobenzoic acid	5 mg/L
thiamine	4 mg/L

## Data Availability

Sequencing data are deposited in the ENA database under accession number PRJEB113194.

## Supplementary Materials

The following supporting information can be downloaded at https://www.mdpi.com/article/10.3390/ijms27219582/s1.
