# Supplementary material for: Gut Microbiota-Mediated SN-38G Metabolism In Vitro: Effects of Murraya koenigii and Ruta graveolens
Source: Int J Mol Sci. 2026 Oct 27;27(21):9582. doi: 10.3390/ijms27219582 (PMC13763257; doi:10.3390/ijms27219582)
Supplement: Supplementary file 1 [file ijms-27-09582-s001.zip › ijms-4558386-supplementary.pdf]

Supplementary Figures and Tables

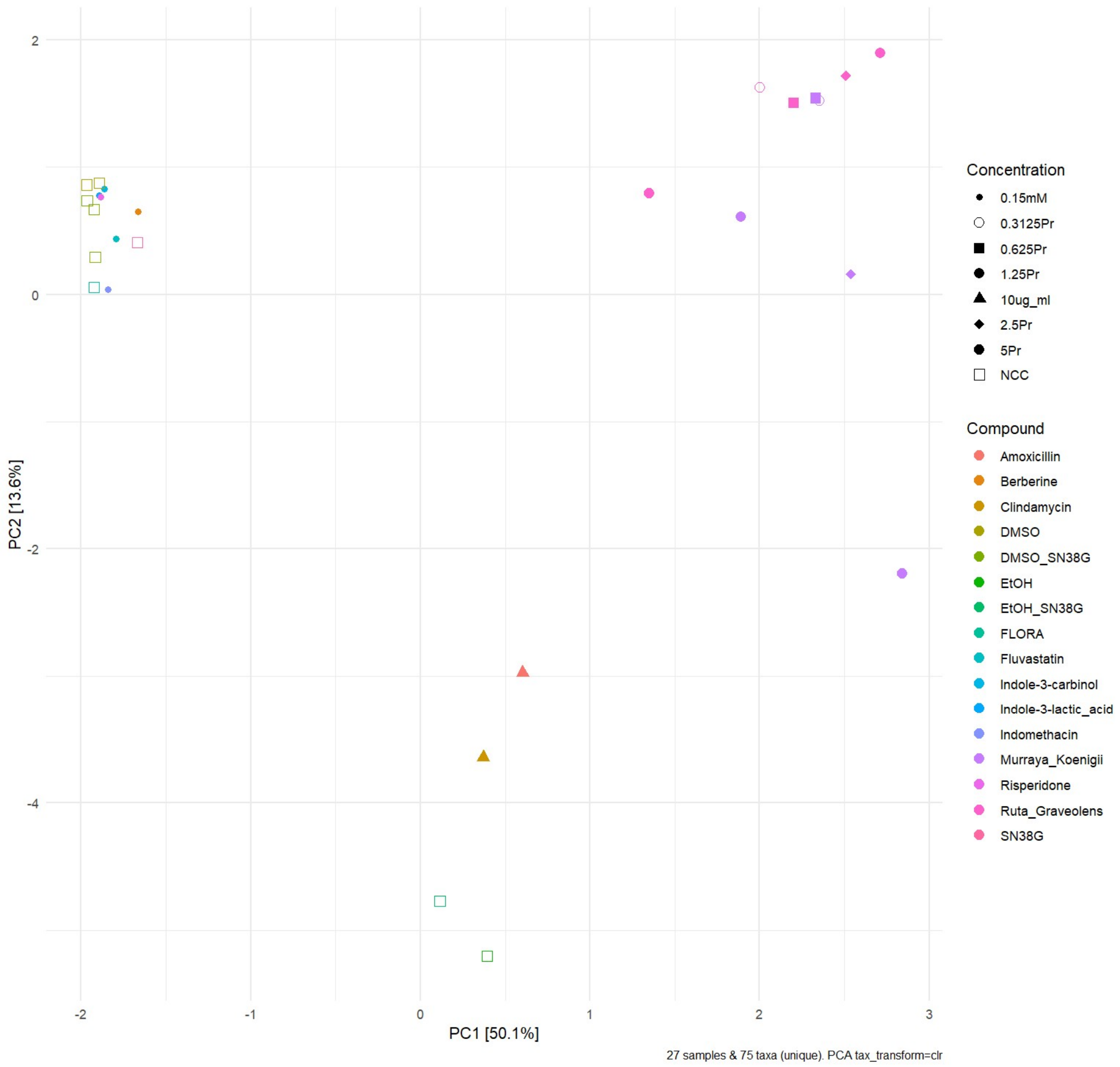

Supplementary Figure S1. Microbiota composition across control, compound, antibiotic and plant-extract conditions.

Supplementary Table S1. Raw data on which Figure 1 is based. Numbers for SN38G and SN-38 represent the percentage compound measured.

Time (hours)	SN-38G			Mean	SD	SN-38			Mean	SD
0	92	93	96	93.67	2.08	6	7	7	6.67	0.58
1	102	79	85	88.67	11.93	14	9	10	11.00	2.65
2	89	81	76	82.00	6.56	18	18	18	18.00	0.00
3	83	78	73	78.00	5.00	25	22	20	22.33	2.52
4	59	52	74	61.67	11.24	37	29	49	38.33	10.07
5	6	7	7	6.67	0.58	90	96	94	93.33	3.06
6	0	0	0	0.00	0.00	97	101	101	99.67	2.31
7	0	0	0	0.00	0.00	101	103	96	100.00	3.61
8	0	0	0	0.00	0.00	126	67	106	99.67	30.01

Supplementary Table S2. Structural overlaps with SN-38G as measured with MicrobeFDT database.

#ID	Name	r-score
60837	IRINOTECAN HYDROCHLORIDE Trihydrate	0.87
60699	TOPOTECAN HYDROCHLORIDE	0.84
126968872	IndomethacinAcyl-beta-D-glucuronide	0.81
53481014	2,8-Dihydroxyquinoline-beta-D-glucuronide	0.81
196513	6-Hydroxy-5-methoxyindole glucuronide	0.79
196512	5-Hydroxy-6-methoxyindole glucuronide	0.79
3083987	AC1L9DOB	0.78
2733785	Indoxyl glucuronide	0.77
628311	AC1LCFOL	0.77
176456	Rutalinium	0.77
14019526	Dihydroxyfumitremorgin C	0.76
5770	reserpine	0.75
53481648	5-hydroxytryptophol glucuronide	0.75
5319758	Platydesminium	0.75
110635	Tadalafil	0.74
441264	3-hydroxyquinine	0.73
53481645	3-Indole carboxylic acid glucuronide	0.73
11972305	Cappariloside B	0.73
53481645	3-Indole carboxylic acid glucuronide	0.73
8549	Quinine (1)	0.72
441074	quinidine	0.72
159650	Pteleine	0.72
3034034	Quinine (2)	0.72
4480711	AC1NAPOE	0.72

5978	vincristine	0.72
91559	Betanine	0.71
11232900	Cappariloside A	0.71
60779	Vinorelbine tartrate	0.70
180314	Murrayazolinol	0.70
5377686	Flazin	0.70
403923	Fumitremorgin C	0.70
5969616	Moschamine	0.70
3715	indomethacin	0.70
4480716	AC1NAPOT	0.69
71752221	sorafenib glucuronide	0.69
53477725	trans-3-hydroxycotinine glucuronide	0.69
10106139	2-Methoxycanthin-6-one	0.69
3157	doxazosin	0.69
11044132	Graveolinine	0.69
72704	Berberrubine	0.69
5401	terazosin	0.69
107936	gamma-Fagarine	0.68
446155	fluvastatin	0.68
896	Melatonin	0.68
160179	Perlolyrine	0.68
3084224	Oxonantenine	0.68
18986	5-Methoxyindole-3-acetic acid	0.68
641764	N-trans-Feruloyl tryptamine	0.68
151018	5-methoxy-L-tryptophan	0.68
5855402	AC1NYHKT	0.68

2353	Berberine	0.68
4481056	AC1NAQGY	0.68
2092	alfuzosin	0.67
5318825	Koenigine	0.67
83944	Acetaminophen glucuronide	0.67
4893	prazosin	0.67
21125948	Tetrahydroxypentoxyline	0.67
10484893	Diferuloyl-4,4'-biserotonin	0.67
97622	Oxoxylopine	0.67
1864	6-HYDROXYMELATONIN	0.67
638215	18110-87-7	0.67
2585	carvedilol	0.67
6436173	Rifaximin	0.66
10144	Liriodenine	0.66
96932	Xanthaline	0.66
656562	AC1LCV7D	0.66
4369318	AC1N9ZWO	0.66
3398121	139427-57-9	0.66
10626660	Neoacrimarine K	0.66
358022	Ascorbigen A	0.66
10507216	Neoacrimarine H	0.66
22297560	(S)-Annocherine A	0.66
21586648	Gravacridonetriol	0.66
25201824	N-[[3-(b-D-Glucopyranosyloxy)-2,3-dihydro-2-oxo-1H-indol-3-yl]acetyl]aspartic acid	0.66
5317836	Gravacridonediol	0.66
3081700	1'-O-Acetylpaxilline	0.66

5317837	AC1NSW36	0.66
118753620	celecoxib glucronide	0.66
5458181	nikkomycin z	0.66
5459911	bilirubin diglucuronide	0.66
4697	4b-Hydroxy-2-(1-hydroxy-1-methylethyl)-12b	0.66
5319094	Annocherine B	0.66
5318827	Koenine	0.66
10484617	4-[N-(p-Coumaroyl)serotonin-4''-yl]-N-feruloylserotonin	0.66
10357912	Azacridone A	0.66
10239	Oxycinchophen	0.65
10327320	Bismahanine	0.65
4828	pindolol	0.65
5488120	BRN 5581827	0.65
167736	3'-Hydroxypapaverine	0.65
97662	Oxoglaucine	0.65
6438344	Glucuronic acid conjugates of bilirubin	0.65
5331104	Paullone Analog 3	0.65
65981	Repaglinide	0.65
24891366	Na-p-Hydroxycoumaroyltryptophan	0.65
903	N-Acetyl-5-hydroxytryptamine	0.65
10428959	N-Caffeoyltryptophan	0.65
3308	etodolac	0.65
1832	N,N-Dimethyl-5-methoxytryptamine	0.65
97487	Koenimbine	0.65
5319215	AC1NSXSL	0.65
71348	Somatuline (TN)	0.65

24883428	dextrorphan O-glucuronide	0.65
5281850	Rutacridone epoxide	0.65
11099949	Dictyoquinazol C	0.65
278055	Koenigicine	0.65
5458879	p-coumaroylserotonin	0.65
122731	Staphylomycin S	0.65
3035848	Nicotine glucuronide	0.65
3360	Floctafenine	0.65
5389314	SMR000041930	0.65
375143	2,9,9,12-tetramethyl-9a,10,11,12,13,13a-hexahydro-9H-1,12-epoxyindolo[3,2,1-de]phenanthridine	0.65
440806	5-Hydroxyindoleacetylglycine	0.65
5282451	Pitavastatin calcium	0.65
377153	NSC658355	0.64
11184822	Koeniginequinone B	0.64
375151	Mahanine	0.64
637050	Dictyoquinazol A	0.64
11479216	CHEMBL449884	0.64
1833	5-METHOXYTRYPTAMINE	0.64
446156	cerivastatin	0.64
5319962	Murrayacine	0.64
5465778	NSC661203	0.64
5321314	Simulanoquinoline	0.64
10085008	Murrayamine A	0.64
617263	Pyridine, 3-[5-[4-[(3-methyl-2-butenyl)oxy]phenyl]-2-oxazolyl]-	0.64
159983	Trp-fru	0.64
23806798	SAn1_008915	0.64

375148	3,8-dimethyl-3-(4-methylpent-3-en-1-yl)-3,11-dihydropyrano[3,2-a]carbazol-9-ol	0.64
144	5-Hydroxy-DL-tryptophan	0.64
4680	papaverine	0.64
284998	OXOPURPUREINE	0.64
5281849	Rutacridone	0.64
1826	5-Hydroxyindole-3-acetic acid	0.64
16191158	Ambcb11985656	0.64
5154	SANGUINARINE	0.64
5319913	Mukonine	0.64
53481609	Xanthurenate-8-O-beta-D-glucoside	0.64
3085285	Juzirine	0.64
2391	bisacodyl	0.64
4829	Pioglitazone	0.64
97679	Norsanguinarine	0.64
5462516	Telithromycin	0.64
11210019	UNII-50H0N03SQU	0.63
375152	Koenoline	0.63
74688	Hydroxyindoleacetaldehyde	0.63
928446	Pyridine, 3-(5-(p-methoxyphenyl)-2-oxazolyl)-	0.63
11221120	UNII-5PO1WPH92W	0.63
53465569	Mukoline	0.63
5315835	Gravacridonechlorine	0.63
4072580	Isomahanimbine	0.63
5486900	Isogravacridonchlorine	0.63
101711	GNF-PF-4292	0.63
4908	PRIMAQUINE	0.63

14804150	6-Methoxymurrayanine	0.63
10506059	Neoacrimarine J	0.63
14380428	Hallacridone	0.63
10697534	Neoacrimarine I	0.63
10903565	DICTYOQUINAZOL B	0.63
10605443	Fumonisin FP1	0.63
24898872	Clausine L	0.63
16212907	Dihydrocinchonine	0.63
5317838	Gravacridonolchlorine	0.63
10999153	mukonidine	0.63
16131130	Pediocin ach	0.63
5458878	N-Feruloyltryptamine	0.63
191040	AT-308	0.63
3148	dolasetron	0.63
9061	5-Hydroxytryptophol	0.63
5311080	Endomorphin 1	0.63
60823	atorvastatin	0.63
96942	Murrayanine	0.62
375162	(1-(2-Amino-3-(4-methoxyphenyl)propanoyl)-5-(6-(dimethylamino)-9H-purin-9-yl)-3-piperidinyl)methanol	0.62
375144	Currayanine	0.62
94577	Cepharadione A	0.62
11739408	UNII-IG3GV1WE4R	0.62
16143525	No_auto_id	0.62
60822	ATORVASTATIN CALCIUM	0.62
45782784	NP-013381	0.62
2762	cinoxacin	0.62

2757	CINCHONIDINE (1)	0.62
21585658	DAPTOMYCIN	0.62
150885	N-Methylserotonin	0.62
400037	ethyl 4-(2z)-3-(1h-benzimidazol-2-yl)-6-methoxy-2h-chromen-2-ylideneaminobenzoate	0.62
11264975	Koeniginequinone A	0.62
5177836	AC1NPEPD	0.62
5281832	Arborinine	0.62
11764751	Bis-p-coumaroyl-4,4'-biserotonin	0.62
9926066	ACMC-20mrza	0.62
9833444	Sincalide	0.62
6918493	Ambrisentan	0.62
9975970	Murrayanol	0.62
4614	oxaprozin	0.62
53465568	Mukolidine	0.62
10676816	Fumonisin FP3	0.62
101744	CINCHONIDINE (2)	0.62
14702537	Acrimarine H	0.62
96943	Girinimbine	0.62
6602291	LS-187369	0.62
10432278	Murrastifoline F	0.62
323935	78205-48-8	0.62
12555	BENZYDAMINE	0.62
10629162	Fumonisin FP2	0.62
336321	Ribaline	0.62
5202	serotonin	0.62
10465540	Phytoalexin	0.62

20725	6,7-Dimethoxy-1-methylisoquinoline	0.62
15394713	1-(1,3,4-Trihydroxybutyl)-beta-carboline	0.62
5281842	Furofoline I	0.62
5280953	HARMINE	0.62
53481655	Alanyl tryptophan	0.62
6695	Rhodamine B cation	0.62
167963	Mahanimbine	0.62
2583	CARTEOLOL	0.61
16132283	Glucagonum	0.61
10009398	Janthitrem F	0.61
11660079	Junosidine	0.61
119405	5,6-Dihydroxy-1H-indole-2-carboxylic acid	0.61
99957	66584-72-3	0.61
6323490	RIFABUTIN	0.61
60934	Saquinavir mesylate	0.61
3496496	AC1MRPAC	0.61
92904	Indole-3-lactic acid	0.61
5315739	N-Acetyldehydroanonaine	0.61
181538	6-Acetonyldihydrosanguinarine	0.61
53481562	HMDB12965	0.61
286538	2-(6-hydroxy-1H-indol-3-yl)acetamide	0.61
5311489	Tryptase	0.61
40692	mefloquine	0.61
14463134	5-Methoxynoracronycine	0.61
5318015	Heptaphylline	0.61
263426	1-methyl-2,3,49-tetrahydro-1h-|A-carboline-1,3-dicarboxylic acid	0.61

69131015	Isoquinoline,6,7-dimethoxy-1-methyl-, 2-oxide	0.61
11024779	Avenanthramide 2s	0.61
50224	Buserelin acetate	0.61
383413	Octreotide (acetate)	0.61
356087	2(1H)-Quinolinone, 3-(2,3-dihydroxy-3-methylbutyl)-4-methoxy-1-methyl-	0.61
17766220	No_auto_id12	0.61
15767846	7-Hydroxyheptaphylline	0.61
10394234	Janthitrem G	0.61
5487591	Citracridone-I	0.60
5494828	AC1NUVSX	0.60
456201	ketoconazole (2R,4S)	0.60
77990	Rivastigmine tartrate	0.60
504068	Mukonal	0.60
16748209	4-Oxopyrimidine, 29a	0.60
115237	Paliperidone	0.60
16131215	Abarelix	0.60
73530	5470-37-1	0.60
5494827	Citpressine I	0.60
119406	4837-74-5	0.60
19093029	Citibrasine	0.60
6323497	RIFAPENTINE	0.60
10531	dihydroergotamine	0.60
1978	acebutolol	0.60
6438400	heme O	0.60
22297200	1-(2-Hydroxyphenylamino)-1-deoxy-beta-D-gentiobioside 1,2-carbamate	0.60
151228	4-Pyridoxolactone	0.60

216239	Sorafenib	0.60
657180	Leuprolide acetate	0.60
11972307	No_auto_id11	0.60
4583	ofloxacin	0.60
10087955	Avenanthramide B	0.60
176870	Erlotinib	0.60
4544	Gnoscopine	0.60
151564	59132-30-8	0.60
9576416	Neoglucobrassicin	0.60
53480450	L-Epinephrine glucuronide	0.60
21593830	3,5-dioxa-11-azahexacyclo[9.9.2.0	
10331015	CHEMBL484460	0.60
7124	Quinoline-2-carboxylic acid	0.60
53481605	HMDB13072	0.60
14039008	Piperolactam D	0.60
53481604	Somatostatin fragment 3-14	0.60
184116	Aristolodione	0.60
92425	Flumioxazin	0.60
149096	LEVOFLOXACIN	0.60
12309935	Fumigaclavine A	0.60
2019	actinomycin D	0.60
259218	5-Methoxycanthin-6-one	0.60
5321000	AC1NT02Z	0.60
41744	Valrubicin	0.60
15394712	1-(1,4-Dihydroxybutyl)-beta-carboline	0.60
47576	ketoconazole (2S,4R)	0.60

10532237	Bis(5-hydroxynoracronycine)	0.60
5458213	Rifampicin	0.60
275196	noscapine	0.60
11052947	2-methoxy-3-methyl-9H-carbazole	0.60
5322012	GROSSAMIDE	0.60
85880351	2,3-Dihydro-3,5-dihydroxy-2-oxo-1H-indole-3-acetic acid	0.60
11167602	regorafenib	0.59
5487772	Citrusinine I	0.59
114683	5,6-Dihydroxyindole	0.59
29979359	S-Equol7-glucuronide	0.59
16132265	corticotropin	0.59
5582	AC1L1KO2	0.59
98285	6052-68-2	0.59
11574561	2-octadecyl-1H-indol-3-ol	0.59
1024	methoxatin	0.59
123631	Gefitinib	0.59
21590181	[33'-Bi-1H-indole]-5,5',6,6'-tetrol	0.59
3081016	Piperolactam A	0.59
3911	Enantone (TN)	0.59
124075	Casimiroin	0.59
5199636	2-(2-CARBOXYACETAMIDO)-3-(1H-INDOL-3-YL)PROPANOIC ACID	0.59
5281838	Buntanine	0.59
5315364	Bicyclomahanimbine	0.59
105068	Pyridinoline	0.59
5315354	No_auto_id0	0.59
5317559	Gentiatibetine	0.59

227613	ASPIDOSPERMINE	0.59
10103438	6-hydroxy-1H-indole-3-carbaldehyde	0.59
230608	5466-34-2	0.59
504069	Methyl 9H-carbazole-3-carboxylate	0.59
10143380	No_auto_id9	0.59
10332932	Dehydro-4-methoxycyclobrassinin	0.59
2179	amsacrine	0.59
10408200	1-(2-hydroxy-3-methyl-9H-carbazol-1-yl)-3-methyl-9H-carbazol-2-ol	0.59
69867	Indole-3-carboxylic acid	0.59
10371106	No_auto_id8	0.59
249630	NSC68131	0.59
10460648	No_auto_id7	0.59
10348646	Janthitrem E	0.59
10468408	Pummeline	0.59
10432264	Bikoeniquinone A	0.59
3082490	Dopamine glucuronide	0.59
161899	Dehydroroemerine	0.59
10947890	Avenanthramide 1s	0.59
441674	Cyanidin 3-O-rutinoside	0.59
24847866	No_auto_id6	0.59
164173	7-Chloro-4-(2-(trifluoromethyl)phenoxy)quinoline	0.59
25074886	Cetrorelix trifluoroacetate	0.59
11010948	4-Methoxybrassinin	0.59
76972097	UNII-82L743IJNX	0.59
71391107	1H-Indole-3-acetic acid,2,3-dihydro-5-hydroxy-2-oxo-methyl ester	0.59
76966715	UNII-P0PUY7ZN10	0.59

441907	Avenacin A-1	0.59
57357311	TANAKINE	0.59
16129617	Cortrosyn (TN)	0.59
72626	Cinnabarin	0.59
44256839	Peonidin 3-sambubioside	0.59
353479	NSC601904	0.59
353825	Graveoline	0.59
10881419	piperolactam C	0.59
5459241	Narceine imide	0.59
5494824	Grandisine III	0.59
166548	Anidulafungin	0.59
5377412	NSC681019	0.59
21674154	Pinotin A	0.59
25077179	D-Tryptophan-LH-RH	0.59
5321315	Simulansine	0.59
25200809	2-[2-(1H-INDOL-3-YL)ACETAMIDO]PENTANEDIOIC ACID	0.58
5492231	Tulipanin	0.58
25202817	kinetin-7-N-glucoside	0.58
25203435	kinetin-9-N-glucoside	0.58
3712	Indole-3-carbinol	0.58
5593	tropicamide	0.58
77999	rosiglitazone	0.58
449193	Roflumilast	0.58
78300	5-Pyridoxolactone	0.58
5488811	AC1NUQWS	0.58
115255	Estrone 3-glucuronide	0.58

9681	methysergide	0.58
6441439	AC1O5VB8	0.58
3080759	Triiodothyronine glucuronide	0.58
11968794	Nelumboside	0.58
44256840	Peonidin 3-sophoroside	0.58
468595	Posaconazole	0.58
6324770	ulfate (2:1)	0.58
6443140	86321-24-6	0.58
386679	NSC679206	0.58
386800	NSC679508	0.58
3830888	MALVIN	0.58
3568969	AC1MT2OY	0.58
200631	2-Hydroxy-1-(1H-indol-3-yl)ethanone	0.58
150345	9-amino-n-(2-(4-morpholinyl)ethyl)-4-acridinecarboxamide	0.58
390235	NSC687527	0.58
6325654	AC1O3DOL	0.58
9604652	Pyriminobac-methyl	0.58
9804518	ZINC13434531	0.58
9601691	Glucobrassicin	0.58
11972447	OJZWDOKDQBCANR-ZPSAUEBXSA-	0.58
21594268	Peonidin 3-(2-(6-(E)-caffeoyl-beta-D-glucosyl)-beta-D-glucoside) 5-beta-D-glucoside	0.58
4480714	AC1NAPON	0.58
171161	AFMK	0.58
11988264	Estradiol-17alpha 3-D-glucuronoside	0.58
9815515	5'-hydroxycotinine	0.58
49844484	1065272-10-7	0.58

25087690	No_auto_id1	0.58
5281114	AC1NQY1K	0.58
12302249	Pelargonidin-3-glucoside (M9)	0.58
4999763	AC1NM8KF	0.58
31100	BROMOCRIPTINE MESYLATE	0.58
16130957	Ganirelix	0.58
6450130	139906-05-1	0.58
4481259	AC1NAQXM	0.58
11169452	Cyanidin 3-O-sophoroside	0.58
22297293	7-Hydroxy-4-methylphthalide O-[arabinosyl-(1->6)-glucoside]	0.58
12313913	No_auto_id2	0.58
10100906	Delphinidin 3,5-diglucoside	0.58
53462879	FT-0698262	0.58
129280	6-Oxocannabidiol glucoside	0.58
10016895	citrusinine-II	0.58
22833607	Mesohydroxy uroporphyrin III	0.58
10245047	Avenanthramide E	0.58
36450	BRN 0504029	0.58
22833525	3-Methoxy-4-hydroxyphenylglycol glucuronide	0.58
12309937	(-)-Fumigaclavine B	0.58
53481380	2-carboxymethyl-3-hydroxyphenylpropionyl-CoA	0.58
13915667	5,7-dihydroxy-3-[3,4,5-trihydroxy-6-(hydroxymethyl)oxan-2-yl]oxy-2-(3,4,5-trihydroxyphenyl)-1$l	
13783824	17-beta-estradiol-3-glucuronide	0.58
160638	Estriol-3-glucuronide	0.58
11972448	No_auto_id3	0.58
5312125	Silodosin	0.58

439862	AC1L986U	0.58
14311149	Petunidin 3-galactoside	0.58
3084569	Sambicyanin	0.58
22954533	Omphalotin C	0.58
23724704	Pelargonidin 3-O-sophoroside	0.58
23724697	Cyanidin 3-(3'',6''-dimalonylglucoside)	0.58
607542	3-Methyl-9H-carbazol-1-ol	0.58
5319251	Malvin chloride	0.58
14311158	delphinidin 3-O-(6''-O-malonyl)-beta-D-glucoside	0.58
56671053	3-[(4,5-dihydroxy-3-[3,4,5-trihydroxy-6-(hydroxymethyl)oxan-2-yl]oxy}-6-[(3,4,5-trihydroxy-6-methyloxan-2-yl)oxy]methyl}oxan-2-yl)oxy]-2-(3,4-dihydroxyphenyl)-5,7-dihydroxy-1$l	
22954531	Omphalotin B	0.58
51136360	Naringenin 7-O-beta-D-glucuronide	0.58
5316496	AC1NSUC6	0.58
5353739	Mahanimbilol	0.58
71315012	Curcumin beta-D-glucuronide	0.58
14861214	Peonidin 3-sophoroside 5-glucoside	0.58
47725	Gosereline	0.58
71777476	Hesperetin 7-glucuronide	0.58
622180	Eduline	0.58
441688	Cyanin	0.58
441699	UNII-YM35G4AYEY	0.58
76970196	UNII-2A0M9HOF8Z	0.58
7225	2-Methylbenzoxazole	0.58
5209	Mukoenine A	0.58
76959109	UNII-629O3H07D9	0.58

189151	Cepharadione B	0.58
5318311	12a-Hydroxypachyrrhizone	0.58
190069	(2S,3S,4S,5R,6S)-6-[4-(2-carboxyethyl)-2-methoxyphenoxy]-3,4,5-trihydroxyoxane-2-carboxylic acid	0.58
86127314	LDTMEECXWUGWBZ-UHFFFAOYSA-N	0.58
11263766	1H-Indole-3-acetonitrile, 4-hydroxy-	0.58
25078009	MLS000028833	0.58
66651	17-alpha-estradiol-3-glucuronide	0.58
11671467	Fostamatinib	0.58
9941060	Isopeonidin 3-glucoside	0.58
443651	Petunidin 3-glucoside	0.58
443652	Malvidin-3-glucoside	0.58
443654	Peonidin-3-glucoside	0.58
3004297	NSC643007	0.58
5073	risperidone	0.58
443913	Pelargonidin 3-O-(6-O-malonyl-beta-D-glucoside)	0.58
443917	Pelargonidin 3-O-rutinoside	0.58
193088	Tyramine glucuronide	0.58
444170	Fenugreekine	0.57
16131433	Camelliatannin G	0.57
5465152	NSC641259	0.57
11316354	Daidzein 7-glucuronide	0.57
55463	NEFLUMOZIDE	0.57
54889	MOEXIPRIL HYDROCHLORIDE	0.57
8617	Indole-3-butyric acid	0.57
448812	Eptifibatide	0.57
446569	tetragastrin	0.57

5860263	AC1NYN6X	0.57
8564	NARCEINE	0.57
25171780	1093679-71-0	0.57
5465153	NSC641260	0.57
74706	methyl 2-(1H-indol-3-yl)acetate	0.57
194340	Maysin	0.57
114918	Cinnabarinic acid	0.57
3744	3-Indolepropionic acid	0.57
5488619	CHEMBL444191	0.57
194566	Apimaysin	0.57
25165313	SCHEMBL3739406	0.57
130881	Olmesartan medoxomil	0.57
2541	candesartan	0.57
11304480	No_auto_id4	0.57
119258	Astilbin	0.57
134018	Febuxostat	0.57
98570	Allocryptopine	0.57
3084961	Wogonoside	0.57
29986850	cis-Resveratrol 3-O-glucuronide	0.57
6442291	2''-p-Coumarylastragalin	0.57
11968884	No_auto_id5	0.57
20979907	Rumexoside	0.57
3162	doxylamine	0.57
44257430	Medicarpin 3-O-(6'-malonylgluclside)	0.57
6453452	Engeletin	0.57
6290103	FLAVONE DERIV	0.57

21159150	2''-O-Acetylrutin	0.57
21159149	6-[(2-[5,7-dihydroxy-2-(4-hydroxy-3-methoxyphenyl)-4-oxo-4H-chromen-3-yl]oxy}-4,5-dihydroxy-6-methyloxan-3-yl)oxy]-3,4,5-trihydroxyoxan-2-yl}methyl (2E)-3-(4-hydroxyphenyl)prop-2-enoate	0.57
21159144	Eujambin	0.57
3080714	Callistephin chloride	0.57
44259208	LMPK12112160	0.57
6440437	AC1O5TLI	0.57
6442954	AC1O5Y7O	0.57
101526	Moxifloxacin hydrochloride	0.57
6440605	78214-14-9	0.57
11103	HEMATOPORPHYRIN	0.57

Supplementary Table S3. Structural overlap with compounds metabolized by beta-glucuronidase (EC number 3.2.1.31), identified with the MicrobeFDT database.

ID	Name	r-score
196512	5-Hydroxy-6-methoxyindole glucuronide	0.79
196513	6-Hydroxy-5-methoxyindole glucuronide	0.79
2733785	Indoxyl glucuronide	0.77
83944	Acetaminophen glucuronide	0.67
5459911	bilirubin diglucuronide	0.66
5459911	bilirubin diglucuronide	0.66
3035848	Nicotine glucuronide	0.65
6438344	Glucuronic acid conjugates of bilirubin	0.65
3082490	Dopamine glucuronide	0.59
160638	Estriol-3-glucuronide	0.58
115255	Estrone 3-glucuronide	0.58
193088	Tyramine glucuronide	0.58

22833525	3-Methoxy-4-hydroxyphenylglycol glucuronide	0.58
3080759	Triiodothyronine glucuronide	0.58
11316354	Daidzein 7-glucuronide	0.57
13632905	soyasapogenol B monoglucuronide	NA
6440956	Retinylglucuronide	NA
5491630	Isorhamnetin 3-glucuronide	NA
5280601	Luteolin 7-O-glucuronide	NA
5273285	Resveratrol 3-O-Glucuronide	NA
3531157	3-alpha,20-alpha-dihydroxy-5-beta-pregnane 3-glucuronide	NA
3084023	1-Salicylate glucuronide	NA
3034755	Androstane-3,17-diol glucuronide	NA
470824	Dehydroepiandrosterone 3-glucuronide	NA
443154	SN-38 Glucuronide	NA
443078	Etiocholan-3alpha-ol-17-one 3-glucuronide	NA
443076	2-Methoxy-estradiol-17beta 3-glucuronide	NA
270604	11-Oxo-androsterone glucuronide	NA
168876	Acyl SA glucuronide	NA
167918	Tetrahydroaldosterone-3-glucuronide	NA
161800	Glycyrrhetic Acid 3-O-Glucuronide	NA
160706	Aldosterone 18-glucuronide	NA
154035	p-Cresol glucuronide	NA
152424	Thyroxine glucuronide	NA
151453	Estriol 17-glucuronide	NA
151223	Estriol 3-sulfate 16-glucuronide	NA
137383	Isovalerylglucuronide	NA

131281	Ketoprofen glucuronide	NA
128210	Had-glucuronide	NA
115145	Benzoyl glucuronide (Benzoic acid)	NA
114833	Androsterone glucuronide	NA
108192	Testosterone glucuronide	NA
88111	Valproic acid glucuronide	NA
66424	17beta-Estradiol glucuronide	NA
127448	Octanoylglucuronide	NA
191195	Phenethylamine glucuronide	NA
163959	CHEBI:88735	NA
126511	Lithocholate 3-O-glucuronide	NA
123796	Pregnanediol-3-glucuronide	NA
53480448	Dehydroisoandrosterone 3-glucuronide	NA
24862077	2-Phenylethanol glucuronide	NA
42622727	Cholestane-3,7,12,25-tetrol-3-glucuronide	NA
444791	alpha-D-glucuronic acid	NA
53477755	HMDB02596	NA
53480449	Cholesterol glucuronide	NA
161223	Pglcua	NA
44263360	15-Hydroxynorandrostene-3,17-dione glucuronide	NA
53477753	HMDB02579	NA

Supplementary Table S4. MicrobeFDT candidates associated with *Ruta graveolens* among compounds linked to beta-glucuronidase metabolism (EC number 3.2.1.31). More background information can be found in [15].

ID	Name	r-score	Derived from
3083987	AC1L9DOB	0.78	Alkaloid from the leaves of *Ruta graveolens* (rue). Ribalinium is found in herbs and spices.
176456	Rutalinium	0.77	Alkaloid from *Ruta graveolens* (rue), *Ruta graveolens* sspecies hortensis. Rutalinium is found in herbs and spices.
5319758	Platydesminium	0.75	Alkaloid from *Ruta graveolens* (rue). Platydesminium is found in herbs and spices.
159650	Pteleine	0.72	Alkaloid tentatively identified in cells of *Ruta graveolens* (rue) grown in continuous light in liq. medium. Pteleine is found in herbs and spices.
11044132	Graveolinine	0.69	Alkaloid of *Ruta graveolens* (rue). Graveolinine is found in herbs and spices.
21586648	Gravacridonetriol	0.66	Alkaloid from *Ruta graveolens* (rue). Gravacridonetriol is found in herbs and spices.
5317836	Gravacridonediol	0.66	Alkaloid from the root tissue culture of *Ruta graveolens* (rue). Gravacridonediol is found in herbs and spices.
5317837	AC1NSW36	0.66	Alkaloid from the root of *Ruta graveolens* (rue). Gravacridonediol methyl ether is found in herbs and spices.
5281850	Rutacridone epoxide	0.65	Alkaloid from roots and callus tissue cultures of *Ruta graveolens* (rue).
5465778	NSC661203	0.64	Alkaloid from the callus tissue of *Ruta graveolens* (rue). Rutagravine is found in herbs and spices.
5281849	Rutacridone	0.64	Alkaloid from *Ruta graveolens* (rue). Rutacridone is found in herbs and spices.
5315835	Gravacridonechlorine	0.63	Alkaloid from *Ruta graveolens* (rue). Gravacridonechlorine is found in herbs and spices.
5486900	Isogravacridonchlorine	0.63	Alkaloid from *Ruta graveolens* (rue). Isogravacridonechlorine is found in herbs and spices.

14380428	Hallacridone	0.63	Minor alkaloid from *Ruta graveolens* (rue) tissue cultures. Hallacridone is found in herbs and spices.
5317838	Gravacridonolchlorine	0.63	Alkaloid from *Ruta graveolens* (rue). Gravacridonolchlorine is found in herbs and spices.
375162	(1-(2-Amino-3-(4-methoxyphenyl)propanoyl)-5-(6-(dimethylamino)-9H-purin-9-yl)-3-piperidinyl)methanol	0.62	Constituent of *Ruta graveolens* (rue). Oleanolic acid 3-[rhamnosyl-(1->4)-glucosyl-(1->6)-glucoside] is found in herbs and spices.
5281832	Arborinine	0.62	Alkaloid from *Ruta graveolens* (rue). Arborinine is found in herbs and spices.
336321	Ribaline	0.62	Minor alkaloid from *Ruta graveolens* (rue). (±)-Ribaline is found in herbs and spices.
5281842	Furofoline I	0.62	Alkaloid from the roots of *Ruta graveolens* (rue). Furofoline is found in herbs and spices.
353825	Graveoline	0.59	Alkaloid from *Ruta graveolens* (rue). Graveoline is found in herbs and spices.
5377412	NSC681019	0.59	Alkaloid from the roots *Ruta graveolens* (rue). 1-Hydroxy-3-methoxy-10-methylacridone is found in herbs and spices.
6325654	AC1O3DOL	0.58	Alkaloid from *Ruta graveolens* (rue). 2-[4-(3,4-Methylenedioxyphenyl)butyl]-4(1H)-quinolinone is found in herbs and spices.

Supplementary Table S5. MicrobeFDT candidates associated with *Murraya koenigii* among compounds linked to beta-glucuronidase metabolism (EC number 3.2.1.31). More background information can be found in [15].

ID	Name	r-score	Derived from
180314	Murrayazolinol	0.70	Minor alkaloid from the stem bark *of Murraya koenigii* (curryleaf tree). Murrayazolinol is found in herbs and spices.
5318825	Koenigine	0.67	Alkaloid from the leaves of *Murraya koenigii* (curryleaf tree). Koenigine is found in herbs and spices.
5318827	Koenine	0.66	Alkaloid from the leaves of *Murraya koenigii* (curryleaf tree). Koenine is found in herbs and spices.
10327320	Bismahanine	0.65	Alkaloid from the stem bark of *Murraya koenigii* (curryleaf tree). Bismahanine is found in herbs and spices.
97487	Koenimbine	0.65	Alkaloid from leaves and fruits of *Murraya koenigii* (curryleaf tree). Koenimbine is found in herbs and spices.
5319215	AC1NSXSL	0.65	Alkaloid from leaves of *Murraya koenigii* (curryleaf tree). Mahanimbinine is found in herbs and spices.
278055	Koenigicine	0.65	Alkaloid from the leaves of *Murraya koenigii* (curryleaf tree). Koenigicine is found in herbs and spices.
375143	2,9,9,12-tetramethyl-9a,10,11,12,13,13a-hexahydro-9H-1,12-epoxyindolo[3,2,1-de]phenanthridine	0.65	Alkaloid from the leaves and stem bark of *Murraya koenigii* (curryleaf tree). (±)-Currayangine is found in herbs and spices.
11184822	Koeniginequinone B	0.64	Alkaloid from the stem bark of *Murraya koenigii* (curryleaf tree). Koeniginequinone B is found in herbs and spices.
375151	Mahanine	0.64	Alkaloid from the leaves of *Murraya koenigii* (curry leaf tree). (R)-Mahanine is found in herbs and spices.
5319962	Murrayacine	0.64	Alkaloid from the stem bark of *Murraya koenigii* (curryleaf tree). Murrayacine is found in herbs and spices.

10085008	Murrayamine A	0.64	Alkaloid from the roots of *Murraya koenigii* (curryleaf tree). Murrayamine A is found in herbs and spices.
375148	3,8-dimethyl-3-(4-methylpent-3-en-1-yl)-3,11-dihydropyrano[3,2-a]carbazol-9-ol	0.64	Alkaloid from seeds of *Murraya koenigii* (curryleaf tree). Pyrafoline D is found in herbs and spices.
5319913	Mukonine	0.64	Alkaloid from the stem bark of *Murraya koenigii* (curryleaf tree). Mukonine is found in herbs and spices.
375152	Koenoline	0.63	Alkaloid from the root bark of *Murraya koenigii* (curryleaf tree). Koenoline is found in herbs and spices.
53465569	Mukoline	0.63	Minor alkaloid from roots of *Murraya koenigii* (curryleaf tree). Mukoline is found in herbs and spices.
4072580	Isomahanimbine	0.63	Alkaloid from the leaves of *Murraya koenigii* (curryleaf tree). (+)-Mahanimbicine is found in herbs and spices.
24898872	Clausine L	0.63	Alkaloid from stem bark of *Murraya koenigii* (curryleaf tree). Clausine L is found in herbs and spices.
10999153	mukonidine	0.63	Alkaloid from the stem bark of *Murraya koenigii* (curryleaf tree). Mukonidine is found in herbs and spices.
96942	Murrayanine	0.62	Alkaloid from the stem bark of *Murraya koenigii* (curryleaf tree). Murrayanine is found in herbs and spices.
375144	Currayanine	0.62	Alkaloid from the leaves and stem bark of *Murraya koenigii* (curryleaf tree). Currayanine is found in herbs and spices.
11264975	Koeniginequinone A	0.62	Alkaloid from the stem bark of *Murraya koenigii* (curryleaf tree). Koeniginequinone A is found in herbs and spices.
9975970	Murrayanol	0.62	Alkaloid from seeds of *Murraya koenigii* (curry leaf tree). Murrayanol is found in herbs and spices.
53465568	Mukolidine	0.62	Minor alkaloid from roots of *Murraya koenigii* (curryleaf tree). Mukolidine is found in herbs and spices.

96943	Girinimbine	0.62	Alkaloid from the roots of *Murraya koenigii* (curry leaf tree). Girinimbine is found in herbs and spices.
10432278	Murrastifoline F	0.62	Alkaloid from roots of *Murraya koenigii* (curryleaf tree). Murrastifoline F is found in herbs and spices.
167963	Mahanimbine	0.62	Alkaloid from the fruits and leaves of *Murraya koenigii* (curryleaf tree). (+)-Mahanimbine is found in herbs and spices.
17766220	No_auto_id12	0.61	Alkaloid from the stem bark of *Murraya koenigii* (curryleaf tree). Mukeic acid is found in herbs and spices.
504068	Mukonal	0.60	Alkaloid from the stem bark of *Murraya koenigii* (curryleaf tree). Mukonal is found in herbs and spices.
11052947	2-methoxy-3-methyl-9H-carbazole	0.60	Alkaloid from the seeds of *Murraya koenigii* (curryleaf tree). 2-Methoxy-3-methyl-9H-carbazole is found in herbs and spices.
5315364	Bicyclomahanimbine	0.59	Alkaloid from the leaves of *Murraya koenigii* (curryleaf tree). Bicyclomahanimbine is found in herbs and spices.
5315354	No_auto_id0	0.59	Alkaloid from the leaves of *Murraya koenigii* (curryleaf tree). Bicyclomahanimbicine is found in herbs and spices.
10408200	1-(2-hydroxy-3-methyl-9H-carbazol-1-yl)-3-methyl-9H-carbazol-2-ol	0.59	Alkaloid from roots of *Murraya koenigii* (curryleaf tree). 1,1'-Bis(2-hydroxy-3-methylcarbazole) is found in herbs and spices.
10432264	Bikoeniquinone A	0.59	Alkaloid from roots of *Murraya koenigii* (curryleaf tree) (Rutaceae). Bikoeniquinone A is found in herbs and spices.
607542	3-Methyl-9H-carbazol-1-ol	0.58	Alkaloid from the stem bark of *Murraya koenigii* (curryleaf tree). 1-Hydroxy-3-methyl-9H-carbazole is found in herbs and spices.
5353739	Mahanimbilol	0.58	Alkaloid from *Murraya koenigii* (curryleaf tree). Mahanimbinol is found in herbs and spices.
5209	Mukoenine A	0.58	Alkaloid from stem bark and roots of *Murraya koenigii* (curryleaf tree). Mukoenine A is found in herbs and spices.

Supplementary Table S6. Relative percentages of SN38G and its metabolite SN38 upon inhibitor response based on LC–MS peak responses.

**Compound**	**SN38G (%)**	**SN38 (%)**
Control	18	82
Berberine 0.05 mM	51	49
Berberine 0.15 mM	68	32
Fluvastatin 0.05 mM	53	47
Fluvastatin 0.15 mM	62	38
Indomethacin 0.05 mM	58	42
Indomethacin 0.15 mM	61	39
MKW 3%	72	28
MKW 10%	76	24
MKE 3%	70	30
MKE 10%	74	26
RGC 3%	80	20
RGC 10%	79	21
RGE 3%	81	19
RGE 10%	87	13

Note. Values represent single-replicate measurements and are reported as the relative percentages of the measured LC–MS peak responses for SN38G and its metabolite SN38. Because no analytical standards were available, these percentages reflect relative peak responses rather than absolute concentrations. LC–MS, liquid chromatography–mass spectrometry.

Supplementary Table S7.

A. Relative percentages of SN38G upon non-treated microbiota fermentation based on LC–MS peak responses

**Time (hours)**	**D38**	**E41**	**E42**	**E43**	**E44**	**E45**	**E46**	**E47**	**E48**	**E49**	**Pool**	**No microbiota**
0	100.00 (0.00)	100.00 (0.00)	100.00 (0.00)	100.00 (0.00)	100.00 (0.00)	100.00 (0.00)	100.00 (0.00)	100.00 (0.00)	100.00 (0.00)	100.00 (0.00)	100.00 (0.00)	100.00 (0.00)
3	37.00 (1.00)	28.33 (2.08)	57.33 (2.08)	38.00 (1.00)	64.00 (4.58)	38.67 (1.53)	60.00 (5.29)	41.33 (0.58)	44.33 (3.06)	64.33 (5.51)	77.00 (8.19)	92.13 (1.27)
4	24.33 (3.51)	20.33 (16.86)	47.33 (11.59)	28.67 (4.93)	47.33 (5.51)	28.67 (3.51)	51.33 (5.03)	33.00 (2.00)	34.67 (6.11)	56.00 (13.11)	70.67 (9.07)	92.02 (0.61)
6	9.33 (7.77)	14.00 (2.65)	40.00 (9.54)	20.67 (1.15)	29.67 (3.21)	22.00 (1.00)	43.67 (4.51)	24.00 (3.46)	27.67 (1.15)	51.67 (2.52)	58.33 (3.79)	93.38 (0.52)
7	4.67 (7.23)	10.33 (0.58)	33.00 (0.00)	14.00 (1.00)	22.33 (0.58)	15.67 (0.58)	34.67 (2.52)	19.33 (1.53)	20.33 (0.58)	46.33 (4.51)	46.67 (5.51)	91.81 (1.17)
8	3.67 (6.35)	6.33 (0.58)	24.00 (0.00)	7.67 (1.53)	14.00 (1.00)	9.00 (1.00)	26.67 (1.53)	14.00 (1.00)	12.33 (0.58)	41.33 (2.52)	33.67 (1.53)	92.86 (0.25)
24	1.00 (1.73)	0.00 (0.00)	0.00 (0.00)	0.00 (0.00)	0.00 (0.00)	0.00 (0.00)	0.00 (0.00)	0.00 (0.00)	0.00 (0.00)	0.00 (0.00)	0.00 (0.00)	89.48 (1.38)

Note. Values are presented as mean (SD) of triplicate measurements for each individual microbiota and represent the relative percentages of the measured LC–MS peak responses for SN38G and its metabolite SN38. No statistical analyses were performed for this table. Because no analytical standards were available, these percentages reflect relative peak responses rather than absolute concentrations. LC–MS, liquid chromatography–mass spectrometry; SD, standard deviation.

B. Relative percentages of SN38G upon clindamycin treated microbiota fermentation based on LC–MS peak responses

Time (hours)	D38	E41	E42	E43	E44	E45	E46	E47	E48	E49	Pool	No microbiota
0	100.00 (0.00)	100.00 (0.00)	100.00 (0.00)	100.00 (0.00)	100.00 (0.00)	100.00 (0.00)	100.00 (0.00)	100.00 (0.00)	100.00 (0.00)	100.00 (0.00)	100.00 (0.00)	100.00 (0.00)
3	43.50 (0.71)	26.50 (0.71)	58.50 (2.12)	37.00 (0.00)	67.50 (7.78)	37.00 (0.00)	61.50 (0.71)	41.50 (2.12)	45.00 (2.83)	65.00 (2.83)	78.50 (3.54)	91.89 (1.69)
4	34.00 (8.49)	21.00 (2.83)	49.50 (6.36)	29.50 (3.54)	59.50 (10.61)	28.00 (2.83)	52.50 (6.36)	33.50 (3.54)	35.50 (0.71)	56.50 (2.12)	72.00 (5.66)	92.09 (0.84)
6	30.50 (0.71)	15.50 (0.71)	48.50 (2.12)	24.50 (0.71)	57.00 (2.83)	23.00 (0.00)	50.00 (1.41)	30.00 (1.41)	32.50 (3.54)	54.50 (6.36)	69.50 (3.54)	93.38 (0.74)
7	25.00 (0.00)	13.50 (0.71)	39.50 (0.71)	21.00 (0.00)	51.50 (3.54)	18.00 (0.00)	44.50 (0.71)	25.00 (0.00)	28.00 (1.41)	49.00 (1.41)	66.00 (8.49)	92.41 (0.75)
8	23.50 (0.71)	11.50 (0.71)	38.00 (1.41)	18.50 (2.12)	48.50 (4.95)	14.50 (0.71)	40.00 (1.41)	21.50 (0.71)	24.00 (1.41)	44.50 (0.71)	63.50 (2.12)	92.79 (0.32)
24	5.00 (0.00)	2.00 (0.00)	4.00 (0.00)	3.50 (0.71)	23.50 (2.12)	1.00 (0.00)	11.50 (0.71)	4.50 (0.71)	7.00 (1.41)	19.00 (2.83)	32.00 (1.41)	88.83 (1.15)

Note. Values are presented as mean (SD) of duplicate measurements for each individual microbiota and represent the relative percentages of the measured LC–MS peak responses for SN38G and its metabolite SN38. No statistical analyses were performed for this table. Because no analytical standards were available, these percentages reflect relative peak responses rather than absolute concentrations. LC–MS, liquid chromatography–mass spectrometry; SD, standard deviation.

C. Relative percentages of SN38G upon *Murraya koenigii* ethanol extract treated microbiota fermentation based on LC–MS peak responses.

**Time (hours)**	**D38**	**E41**	**E42**	**E43**	**E44**	**E45**	**E46**	**E47**	**E48**	**E49**	**Pool**	**No microbiota**
0	100.00 (0.00)	100.00 (0.00)	100.00 (0.00)	100.00 (0.00)	100.00 (0.00)	100.00 (0.00)	100.00 (0.00)	100.00 (0.00)	100.00 (0.00)	100.00 (0.00)	100.00 (0.00)	100.00 (0.00)
3	54.33 (15.14)	51.00 (14.00)	64.67 (24.17)	47.00 (13.00)	66.00 (17.44)	51.67 (13.58)	66.67 (17.04)	53.67 (14.47)	55.33 (15.31)	69.33 (17.67)	81.67 (23.18)	92.13 (1.27)
4	42.67 (3.51)	40.33 (4.51)	55.67 (40.86)	37.33 (2.52)	58.67 (3.21)	42.00 (3.00)	57.00 (5.20)	43.33 (5.13)	46.00 (7.00)	63.00 (4.00)	78.00 (2.00)	92.02 (0.61)
6	40.67 (3.79)	37.67 (0.58)	54.00 (8.89)	33.67 (1.15)	55.00 (1.00)	38.67 (1.53)	56.00 (1.00)	41.00 (2.65)	42.00 (1.00)	58.67 (0.58)	76.00 (2.65)	93.38 (0.52)
7	35.67 (4.04)	33.00 (1.00)	48.33 (7.57)	29.00 (1.00)	48.67 (0.58)	33.00 (1.00)	49.33 (4.73)	35.33 (1.53)	36.00 (1.00)	53.67 (3.21)	71.67 (4.04)	91.81 (1.17)
8	31.33 (2.08)	30.00 (0.00)	46.33 (34.78)	26.00 (1.00)	46.00 (1.00)	29.67 (2.08)	47.67 (1.53)	32.67 (1.53)	32.33 (0.58)	51.00 (1.00)	70.67 (3.06)	92.86 (0.25)
24	9.67 (0.58)	9.00 (1.00)	15.67 (2.89)	6.00 (0.00)	13.00 (2.00)	7.00 (0.00)	13.33 (3.79)	8.67 (1.53)	7.33 (0.58)	20.33 (0.58)	38.00 (1.00)	89.48 (1.38)

Note. Values are presented as mean (SD) of triplicate measurements for each individual microbiota and represent the relative percentages of the measured LC–MS peak responses for SN38G and its metabolite SN38. No statistical analyses were performed for this table. Because no analytical standards were available, these percentages reflect relative peak responses rather than absolute concentrations. LC–MS, liquid chromatography–mass spectrometry; SD, standard deviation.
